# Global social identity predicts cooperation at local, national, and global levels: Results from international experiments

**DOI:** 10.3389/fpsyg.2023.1008567

**Published:** 2023-06-30

**Authors:** Gianluca Grimalda, Nancy R. Buchan, Marilynn B. Brewer

**Affiliations:** ^1^Kiel Institute for the World Economy, Kiel, Germany; ^2^Darla Moore School of Business, University of South Carolina, Columbia, SC, United States; ^3^Ohio State University, Columbus, OH, United States

**Keywords:** global social identity, cooperation, international experiments, local cooperation, national cooperation, global cooperation, local social identity, national social identity

## Abstract

Individuals who identify themselves with humanity as a whole tend to be more prosocial in a number of different domains, from giving to international charities to volunteering for humanitarian causes. In this paper, we show that global identity is “inclusive” in character. That is, rather than neglecting or diminishing attachments to local and national groups, identification with all of humanity encourages individuals to embrace local and national goals at no lesser intensity than they embrace global goals. We have done so using experimental data on social dilemmas at the local level and nested social dilemmas at the local and national level, as well as at the local and world levels. Experiments were conducted with adult samples in the United States, Italy, Russia, Argentina, South Africa, and Iran. We show that the higher the identification with global collectives, net of identification with local and national collectives, the higher the cooperation at the local, national, and world levels. Conversely, local social identity is not significantly associated with cooperation at any level of interaction, while national social identity, net of local and global identification, tends overall to have a negative correlation with cooperation, particularly at the local level. We also show that individuals with strong global identity are significantly more optimistic of others’ contributions than individuals with lower levels of global identification, but they are as accurate as others in predicting others’ cooperation at the local and national levels. Their forecast error is instead systematically larger than that of all others for cooperation at the world level.

## 1. Introduction

One of the tenets of social psychology is that individuals identify themselves with groups and make group goals their own goals (Kramer and Brewer, 1986; Turner et al., 1987; Brewer, 1991; De Cremer and Van Vugt, 1999). Converging evidence has been gathered in support of the idea that individuals who identify themselves with humanity as a whole, rather than (or in addition to) identifying with their nation or their locality, are also more prosocial in a number of different domains, including support for international charities, volunteering for humanitarian causes, collective action against global injustice, environmental activism, and concern for human rights (Barth et al., 2015; Renger and Reese, 2017; McFarland et al., 2019). Individuals with higher identification with humanity as a whole (IWAH) have been shown to be more willing than others to give to charities, particularly those with global humanitarian goals. This has been shown to be the case in both hypothetical survey questions and actual monetary donation choices. Ethnocentrism, which is attachment to the local community and nation, had a less significant role (McFarland et al., 2012; McFarland, 2017; Hamer et al., 2019; Sparkman and Hamer, 2020).

Using a measure of global social identity that is structurally equivalent to the identity subscale of the IWAH measure, Grimalda et al. (2021) also found that individuals with high social identity were more inclined to donate to charities that are active at the global level in providing COVID-19 relief and were more generous than those who donated at the national or local level. In addition, individuals with high global social identity have been found to be more willing to cooperate at the global level in international cooperation experiments (Buchan et al., 2011).

While this converging evidence suggests that globally identifying individuals are more prosocial than others when interacting at the global level, it leaves open the question as to whether these individuals are more or less prosocial than others when interaction takes place at the lower levels of collectivities—the local and national levels in particular. One could posit that global identity is “exclusive” in character. This would be the case if global identity represents strong psychological identification only with distal others and global-level causes, while at the same time weakening identification with members of local and national communities and diminishing the willingness to support local and national goals. The contrasting hypothesis is that global identification is “inclusive” in character. That is, rather than neglecting or diminishing the attachment to local and national groups, identification with all of humanity may encourage individuals to embrace local and national goals at no lesser intensity than they embrace global goals. What would characterize global social identification, then, would be the propensity to extend the “radius” of prosociality to the highest possible level without abandoning identification with the lower levels.

In this paper, we draw on data collected in previous research by our team (Buchan et al., 2009) to test whether global social identity is exclusive or inclusive in character. In experiments conducted in six different countries using a nested social dilemma paradigm, we were able to assess the role of local, national, and global identity as predictors of contributions to a public good involving local, national, or global collectives. In addition, we evaluated the influence of global identification relative to other measures of globalization and global concerns on cooperation.

The extant literature takes opposing views on the nature of global identity. The theoretical political science literature tends to see national and global identity as mutually exclusive (Smith, 1995; Strange, 1996; see Kymlicka, 2001 for an exception). Identifying with global others entails almost automatically distancing oneself from local or national identification. Thus, the matter of debate is whether globalization fosters one or the other form of identity. Some scholars—most notably Hobsbawm (1992)—have argued that nationalism will progressively wane, because the relentless transnational flow of information, people, and products made possible by globalization (Gygli et al., 2019) reduces the capability for any single national identity to retain its unique significance and distinguish itself from other national identities (Ariely, 2012). Conversely, others—most notably Smith (1995) and Calhoun (2007)—have argued that it is precisely in a time of increased globalization that a sense of national belonging is even more needed. Some have posited the possibility of a “backlash” against globalization in some groups, while other groups, especially elites, would develop global identities (Norris, 2005; Castells, 2011).

The empirical attempts to measure the impact of country-level globalization on identity have obtained contrasting results. While some have found evidence of a negative link between globalization and nationalism (Norris and Inglehart, 2009; Cichocka et al., 2022), others have found no effect (Jung, 2008). Ariely (2012) detects a negative effect of globalization on some aspects of national identity—namely, patriotism and the view that ethnicity defines national identity—but not on others—namely, national identification and the view that one’s national culture is superior to others’ cultures. Hamer et al. (2018) show how IWAH connects with national identification, patriotism, nationalism, and collective narcissism.

The social psychology literature has also investigated the notion of inclusiveness, albeit from a different angle. The matter of the debate has been whether prosociality, i.e., the willingness to cooperate with others, is limited to one’s ingroup—i.e., the group with which an individual identifies—or is universal. Some have posited an exclusive nature of prosociality (Aaldering et al., 2018; De Dreu et al., 2022), which may be driven by evolutionary processes of group-level selection whereby the development of cooperation for one’s ingroup is associated with the willingness to hurt the outgroup (Choi and Bowles, 2007). Others have claimed that individuals classified as prosocials, on the basis of either social value orientation or the humility–honesty scale, are recognizably universal in their prosociality (Thielmann and Böhm, 2016; Aaldering and Böhm, 2020). That is, their willingness to cooperate is not limited to one’s ingroup but extends to the outgroup as well.

The main hypothesis we want to test is whether global social identity is inclusive or exclusive in character. In our approach, inclusiveness implies that people identifying with global others should be as cooperative in national and local interactions as they are in global interactions. Exclusiveness implies that people identifying with the global community should be less cooperative in national and local interactions than they are in global interactions. Our previous work (Buchan et al., 2009, 2011) focused exclusively on global cooperation and ascertained that global social identity is highly correlated with cooperation at the global level. Such studies were, therefore, silent on how global social identity correlated with cooperation at the local and national levels. This is the subject of this paper. We find that global social identity is inclusive in character, in that the higher the score in the GSI, the higher the propensity to cooperate at the national and local levels.

After investigating the nature of the relationship between GSI and cooperation, we wanted to better understand the psychological mechanisms underpinning this relationship. It has been posited that expectations on others’ cooperation have an important role in shaping individual propensity to cooperate (Brewer, 1986; Yamagishi and Kiyonari, 2000; Foddy et al., 2009). We have posited a theoretical mechanism whereby social identity, at various levels, influences expectations on others’ behavior at the same level, which in turn affects cooperation. We test this hypothesis through a Sobel–Goodman mediation analysis.

Finally, after establishing the relevance of expectations as a mediator between GSI and cooperation, we investigate whether the expectations are accurate or misplaced. Uslaner (2002) and Yamagishi (2007) posit that trusting individuals may be excessively optimistic of others’ trustworthiness, thus leading to levels of trust in others that are, in fact, economically unprofitable. A process of cognitive dissonance (Festinger, 1954) may keep up members’ motivation to trust others even if these others are not entirely trustworthy. An alternative account is that trusting individuals does hold realistic beliefs on others’ low trustworthiness levels, their trust in others being supported by their altruism rather than by their expectations. In relation to this, Dorrough and Glöckner (2016) find large inaccuracies in the way individuals estimate cooperation levels among people from different countries. Comparing our measures of expectations on others’ cooperation in the experiment with actual cooperative behavior, we ascertain that individuals scoring high on GSI are somewhat overly optimistic on others’ global cooperation levels but are as accurate as others in their expectations at the local and national levels.

## 2. Methods

The experimental protocol, instructions, and questionnaire are available in the Appendix: Supplementary Methods and at the project repository: https://osf.io/ks2u5.

### 2.1. Sample

Our project involved adult populations from specific locations in six different countries (Iran, South Africa, Argentina, Russia, Italy, and the United States). Research sites were selected for this research with the goal of representing a sufficient degree of variability on the globalization spectrum as ranked by the Country Globalization Index (CGI), developed by Lockwood and Redoano (2005) (see Appendix: Section A.1 and Table A.1 for details on the CGI index). Six countries were chosen, with the aim of both maximizing the dispersion of each sphere of the CGI—namely, the economic, social, and political spheres—and of ensuring a sufficient geographic dispersion, so that each continent other than Oceania was represented. The resulting countries were Italy and Argentina (at the highest and lowest positions in the economic globalization subindex, respectively); United States and South Africa (at the extremes of the social globalization index); and Russia and Iran (at the extremes of the political globalization index).

We selected several locations in each country that, on the basis of available information prior to conducting the research, represented differing levels of exposure to globalization in terms of, for instance, the relative presence of multi-national corporations or the presence of immigrant populations. In general, in each country a large urban center was designated as the “hub” of the fieldwork, and less globalized towns or villages were selected within a radius of around 100 miles. Hub localities in the United States, Italy, Russia, and Argentina were Columbus (Ohio), Milan, Kazan (Tatarastan), and Buenos Aires, respectively. For logistical constraints, the same strategy was not feasible in Iran and South Africa. In Iran, the two research sites were Tehran, Iran’s capital and largest city, and Shiraz, the fifth largest city. In South Africa, the research sites were three districts of Northern Johannesburg and the district of Soweto, residents of the latter district being almost exclusively of Black ethnic background. The research sites within Iran and South Africa are, nonetheless, characterized by appreciably different degrees of exposure to globalization within each country, thus ensuring the comparability of our samples across the countries.

Approximately 200 participants were recruited in each country according to a quota sampling method, the aim of which is to target a uniform distribution of observations across relevant demographic dimensions. This method is suitable for cross-country research because it achieves comparability. In our study, the criteria determining the quotas were age (three categories: 19–30, 31–50, and 51–70), gender (two categories: male and female), and social economic status (three categories: high, intermediate, and low). Descriptive statistics by country are reported in the Appendix: Table A.2.

### 2.2. Measurement of cooperation

The participants in our research took part in three experimental decisions that assessed their propensity to cooperate in public goods games (PGG), which entailed cooperation at the local level only (Decision 1), at the local and national level in a nested PGG (Decision 2), and at the local and global level in a nested PGG (Decision 3). Cooperation was measured through a Multi-level Sequential Contribution (MSC) game. The setting is similar to standard PGGs, except that that participants’ decisions were made sequentially rather than simultaneously. The participants’ decisions affected the payoffs of other participants taking part in future sessions. In turn, the participants’ payoffs were determined by their own decisions and by the decisions made by participants in previous sessions.

At each decision stage, every participant was endowed with 10 tokens, each worth the purchasing power equivalent of US $0.50 in each country. In Decision 1, participants could allocate tokens between a “Personal” account and a “Local” group account. Three other individuals from the same locality as the participant also contributed to the Local group account. All tokens allocated to the Personal account were transferred to the individual at the end of the session in their entirety. That is, their marginal *per capita* return (MPCR) was 1.0. The tokens contributed by the four individuals to the Local account would be doubled by the researcher. The participant would then receive a quarter of such a doubled amount. Contributions to the Local account were characterized by the typical properties of a public goods game. First, each token contributed would beget a benefit to others. The Social Return in this case equaled 2.0, because each token contributed was multiplied by two before being returned to the group members. Second, since each token contributed to the group account would only yield half a token in return—resulting in an MPCR of 1/2—an individual desiring to maximize their payoffs should have contributed nothing to the Local account. On the contrary, individuals willing to maximize the total group payoffs should have contributed all 10 tokens.

Decision 2 was a nested PGG in which each participant could allocate the 10 tokens across three different groups named “Personal,” “Local,” and “Country.” Each token allocated to the Personal account again had an MPCR of 1.0 and yielded no benefits to others. Each token allocated into the Local account had the same returns as in Decision 1, that is, an MPCR of 1/2 and Social Returns equal to 2.0. A total of 12 individuals could contribute to the National account—the same four individuals comprising the Local group and two other groups of individuals from other localities in the country. Each token contributed to the National account would be multiplied by three by the researchers. A participant would then receive 1/12 of this amount. The MPCR from contributing to the National account was thus 1/4, lower than the MPCR from contributing to the other accounts. Nevertheless, the Social Returns equaled 3.0 and were thus higher than the Social Returns for the Local and Personal accounts. We believe that this return structure adequately represented the incentives and costs of cooperating at a local vs. a more aggregated level.

Finally, Decision 3 was a nested PGG in which individuals could allocate 10 tokens to the “Personal,” “Local,” and “World” accounts. The World group involved the same four individuals from the Local group and two groups of individuals from other countries. The MPCR and the Social Returns for each account were the same as those for Decision 2 for the corresponding account. Each token allocated to the Personal account again had an MPCR of 1.0 and no Social Returns. Each token allocated into the Local account had an MPCR of 1/2 and Social Returns equal to 2.0. Each token contributed to the World account had an MPCR of ¼ and Social Returns of 3.0. Even in Decisions 2 and 3, an individual desiring to maximize their final payoffs should have contributed nothing. The parameters of the three decisions are reported in Appendix: Table A.3. A summary of the experiment protocol and the whole experiment script are reported in Supplementary Methods: Sections SM.2–3.

The national areas and countries involved in Decision 2 and Decision 3, respectively, were not named. In particular, the participants were informed that these countries might have been in any part of the four continents where the research was conducted. Not naming countries or national areas made choices unaffected by biases or stereotypes about particular nationalities. This is important, because stereotypes can be deeply rooted and widespread worldwide while being simultaneously fundamentally wrong (Dorrough and Glöckner, 2016). This approach is also consistent with a definition of globality as a notion that transcends mere internationalization (Robertson, 1992; Scholte, 2005). Thus, contributing to either the Local or World accounts can be classified as a cooperative act in that the individual sacrifices immediate personal gain for greater gain at the collective level. The participants’ identities were not revealed either to other participants or to the experimenter, as the game was played in conditions of anonymity. The participants were told that they were involved in a series of decisions involving people from their own local area, some of whom may or may not have been in the same room, and from other countries around the world.

**Figure 1:**
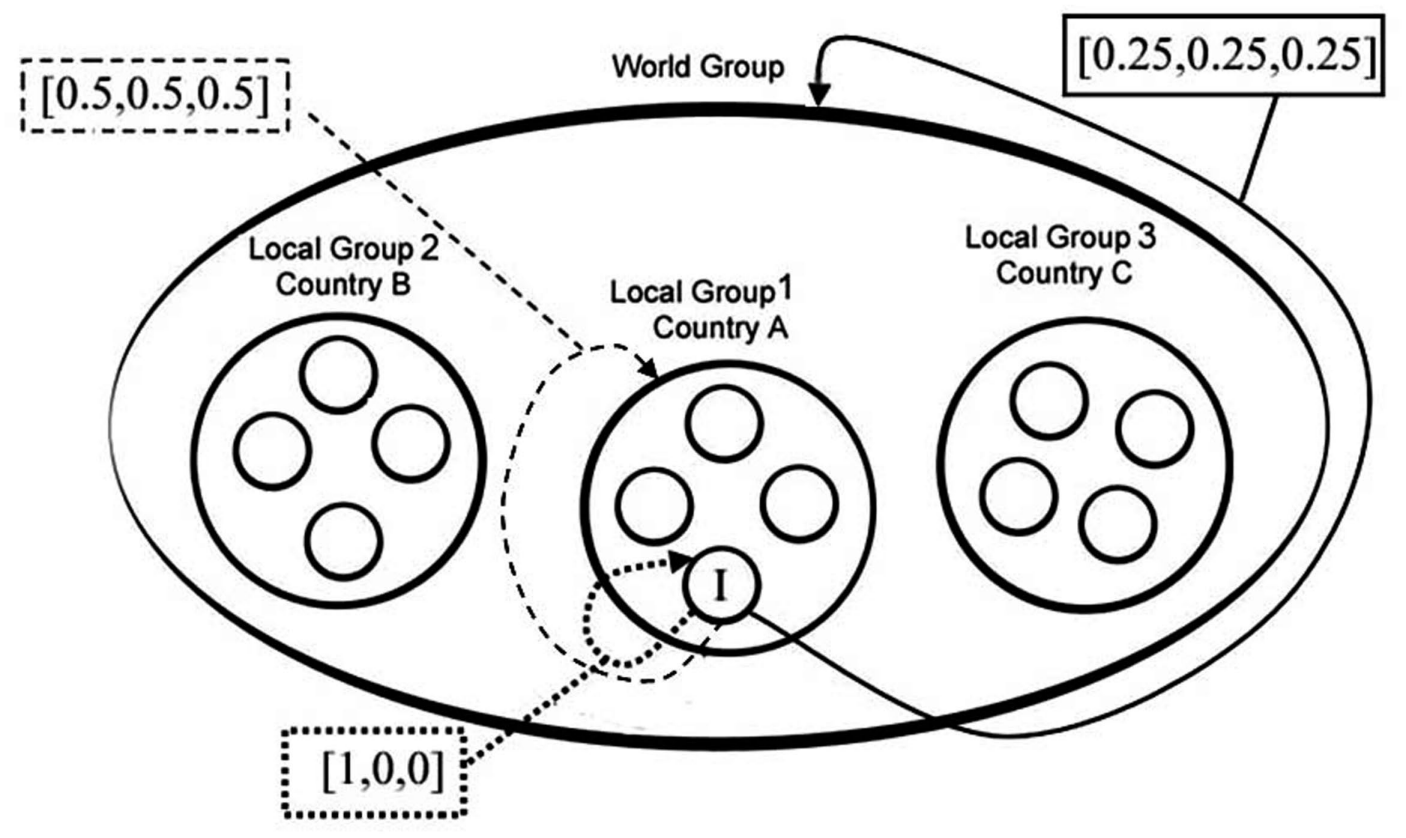
Representation of the nested social dilemma for Decision 3. I denotes the “Individual” participant. “Local 1,” “Local 2,” and “Local 3” represent groups of people residents in the same locality in three different countries. Individuals had three options on how to allocate their endowments of 10 tokens: allocating to a personal account, to their local account, and to the global account, which comprises the three lower-level local accounts. Contributions to the personal account were transferred one-to-one onto an individual’s payoff. Contributions to one’s local account were multiplied by a factor of two and divided among four local residents. Contributions to the global accounts were multiplied by a factor of three and divided evenly among the 12 participants.

The structure of incentives resembled a nested PGG similar to that employed by Wit and Kerr (2002) and Blackwell and McKee (2003). The design is displayed schematically in Figure 1. In the MSC, an individual willing to maximize their final payoffs should have allocated all their tokens to the Personal account, because both the Local and World accounts bore a smaller MPCR. If no one contributed, each participant would take home their initial 10 tokens. In our MSC, there was a tension between individual returns, social returns, and the locality of the people benefitting from one’s contribution. Individuals allotting their tokens to their Local account could ensure the maximization of the interests of the Local constituency. However, if everyone contributed their endowment to their Local account, the final individual payoffs would be 20 tokens, which is less than if everyone allotted their tokens to the World account, that is, 30 tokens.

### 2.3. Measurement of social identity and other relevant constructs

Three social identity measures were included in the post-experiment questionnaire. The items were taken from the measure of social identity constructed by Yuki et al. (2004) and adapted to assess social identification at the levels of the local community, the nation, and the world. For example, in Kazan, Russia, the items measuring social identity at the level of the local community read:

1. How strongly do you feel attachment to *your community in Kazan*?
2. How strongly do you define yourself as a member of *your community in Kazan*?
3. How close do you feel to other members *of your community in Kazan*?

Social identities at the national and global levels, respectively, were measured by substituting “your community in Kazan” with following expressions: *“Russia”* (*or “Russian community”*)*”* and *“the world as a whole.”* Responses to each item were made on a rating scale from 1 (not at all) to 4 (very much).

The questionnaire also included some questions to assess awareness of and attitudes toward global processes. Robertson (1992) suggests that a key aspect of globalization is, in addition to participation in global networks, the “consciousness of the world as a whole.” It is therefore important to assess how the key constructs in our analysis relate to one’s global awareness. We constructed a “Global Awareness Index” based on the answers to four questionnaire items inquiring about a participant’s awareness of the following global issues: global warming, the global spread of potentially dangerous diseases, the action of the International Criminal Courts of justice, and the persistent gap between rich and poor people around the world (see Appendix: Supplementary Methods SM.4, Question 4).

We also measured individual participation in global relations. Analogous to the CGI, this measure was designed to capture individual access to globalization within the social, cultural, political, and economic spheres. The resulting Individual Globalization Index (IGI) is a summative scale of 30 questionnaire items. The IGI index measures an individual’s usage of various global networks in terms of two dimensions: the frequency with which an individual accesses the networks and the territorial scope. The index identifies several media of global connection and measures the temporal frequency with which the medium of connection is used by the individual and whether such a medium is used to contact people at the local, national, or global level. Although a given medium of connection, such as email, has a potentially global reach, an individual can also decide to use it for contacts at the local or national levels. The IGI, therefore, assigns higher scores to individuals who participate in the global network more frequently and to a wider extent than others. Further details on the IGI and the list of items making up the IGI are reported in Appendix: Section A.1 and Supplementary Methods: SM.5.

## 3. Results

Codes to reproduce the analyses and the output of the statistical analyses are available at the project repository: https://osf.io/ytp9s/.

### 3.1. Descriptive statistics

The social identification scores at each level (local social identity—LSI; national social identity—NSI; and global social identity—GSI) were calculated by summing up the responses to the three items described in section 2.3. The scores, given originally in a 1–4 scale, have been normalized to the 0–1 interval. Thus, individuals scoring one (zero) in, for example, LSI answered that they felt a very strong attachment (no attachment) to their local community, defined themselves very strongly (not at all) as a member of their local community, and felt very close (not close at all) to other members of their local community. Individuals who expressed intermediate levels of attachment/membership/closeness (options 2 and 3 in the original scales) scored in the interior of the (0, 1) interval (see Appendix: SM4 Research Questionnaire, questions 21–23). The Cronbach’s alphas for the indexes at the country level and in the aggregate are reported in Appendix: Table A.4. The alphas are always greater than 0.70, suggesting that the indexes are reliable, except for Russia, for which the alphas are around 0.60. A principal component analysis, however, suggests the unidimensionality of the three indexes, even for Russia (see analyses output).

**Figure 2:**
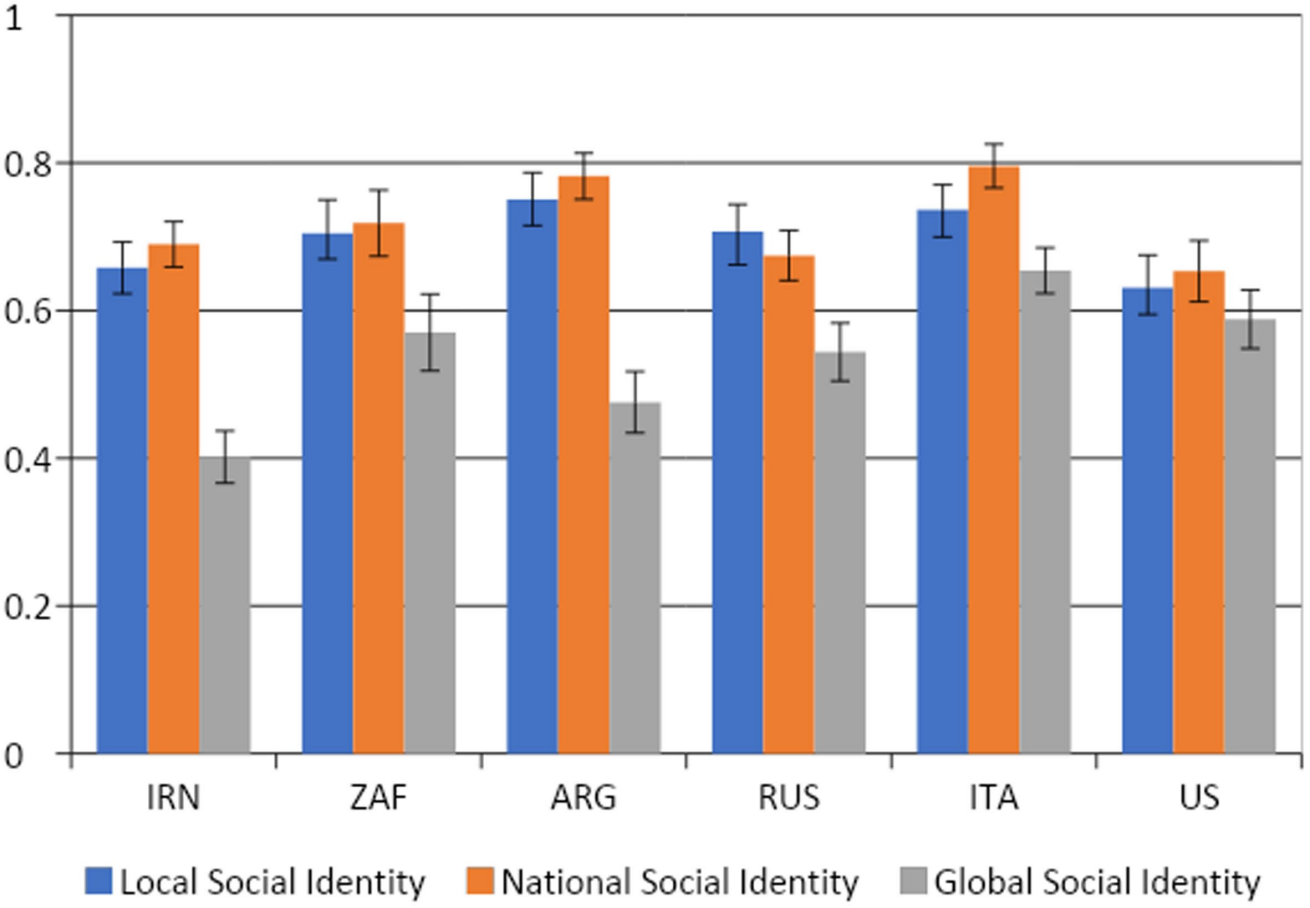
Mean of local, national, and global social identity by country. Capped bars plot 95% confidence intervals for the mean.

Figure 2 plots the means of the three social identity measures in each country. For all countries, except the Russia, the strongest identification was on average at the national level, followed by the local and then the global levels. In Russia, identification was strongest at the local level, followed by the national and the global levels. The analyses of the differences in the social identity indexes through non-parametric tests are reported in Appendix: Section A.2.

As mentioned in the introduction, our measure of global social identity is structurally equivalent to the identity items of the McFarland et al. (2012) “Identification with all humanity” (IWAH) measure that evaluates the extent to which an individual “cares for all humanity, not just for their ingroups.” In the IWAH, respondents are asked to evaluate their identification with and attitudes toward (a) people in their community, (b) conationals, and (c) “all humans everywhere.” Although the phrasing used to identify these three categories differs slightly from the one used here, the two measures appear comparable. In a sample comprising US participants only, the IWAH measure showed the same pattern we found in our study, with identification with the global community being lower than identification with local and national communities, the latter two being approximately equal to each other. Our multi-national analysis enables us to state that this same pattern holds even more pronouncedly in other countries, given that the US was at the lower end of the differences between GSI and the other social identity measures.

**Figure 3:**
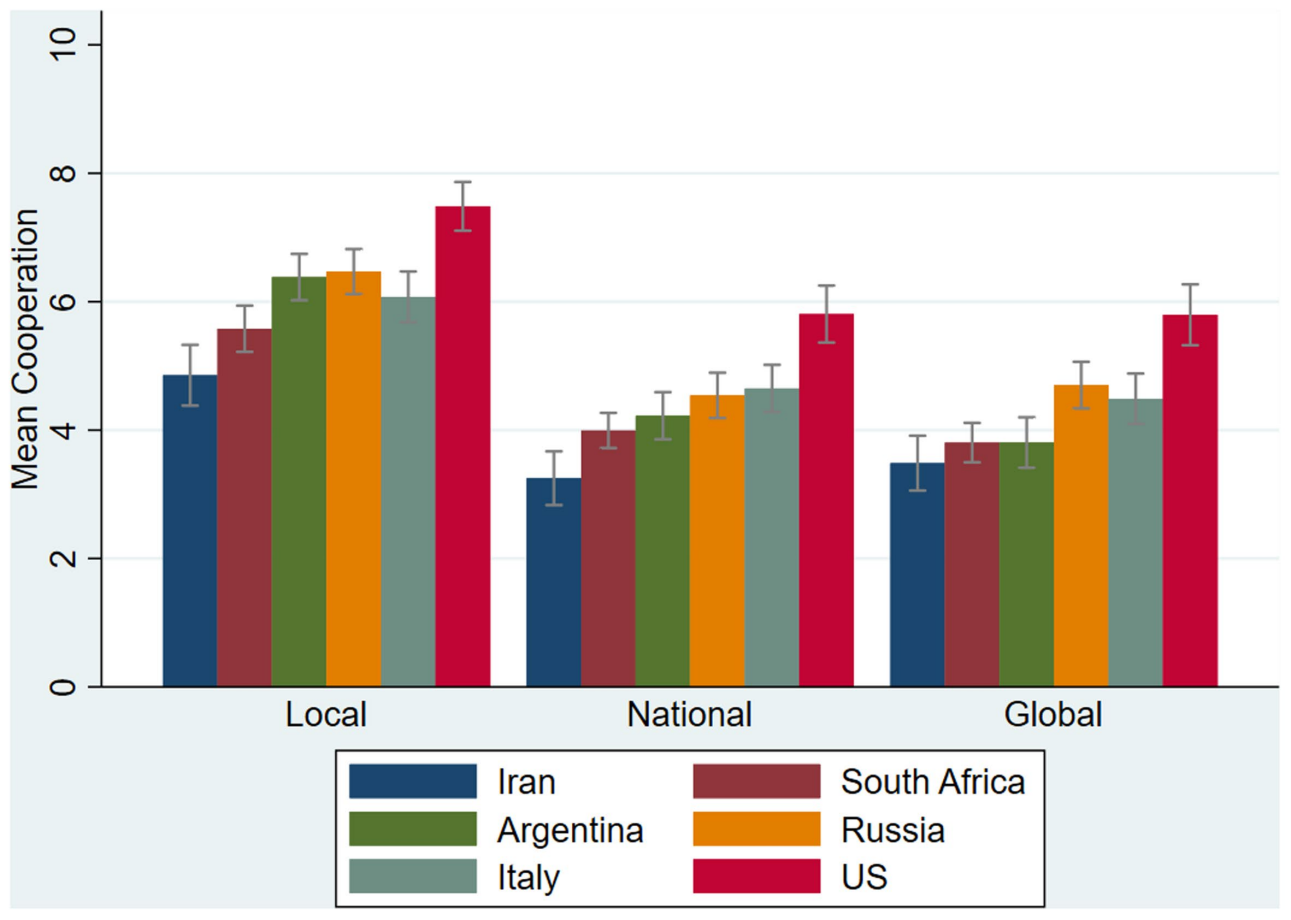
Mean cooperation by decision and country. Capped bars denote 95% confidence intervals of the mean.

The Figure 3 plots mean cooperation according to the decision and country. One can note that cooperation in the first decision (local) is higher than cooperation in the second (national) and third (global) decisions. This is a consequence of the first decision being non-nested, so that individuals only had one public account to give to rather than two public accounts in the second and third decisions. Countries were ranked according to their globalization level, as measured by the CGI (see section 2.1). It is noticeable that mean cooperation tended to increase with the level of globalization of a country. This pattern extends what was already reported in Buchan et al. (2009), though for the third decision only. We report the correlations between social identity measures and cooperation in Appendix: Table A.5.

**Figure 4:**
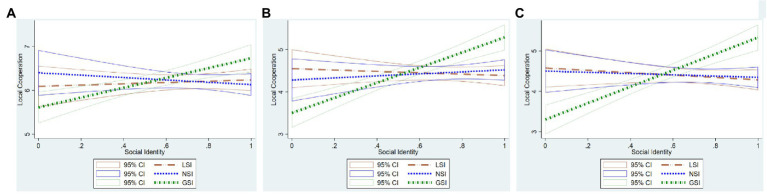
Plots of prediction of **(A)** local cooperation, **(B)** national cooperation, and **(C)** global cooperation from linear regressions on local social identity (LSI), national social identity (NSI), and global social identity (GSI). The solid lines plot a 95% confidence interval of the mean.

Figure 4 plots the prediction of cooperation at each of the three levels based on a simple bivariate OLS regression in which the three levels of social identity enter as independent variables. It is evident that GSI tends to have a markedly positive slope in all three decisions, particularly in the national and global decisions. Both LSI and NSI do not appear to be significantly correlated with cooperation at any level.

### 3.2. Econometric analysis

#### 3.2.1. Analysis of relationship between global social identity and cooperation

We tested the hypotheses of the inclusivity vs. exclusivity of global social identity through Tobit regressions with dependent variables (DVs), those being cooperation at the local level in Decision 1 (Appendix: Table A.6), cooperation at the national level in Decision 2 (Appendix: Table A.7), and cooperation at the world level in Decision 3 (Appendix: Table A.8). We used a Tobit model to account for the truncated nature of the propensity to cooperate in the experimental decision (see Appendix: Section A.3 for a description of the Tobit model). Standard errors are clustered at the session level to avoid heteroschedasticity (White, 1980; Abadie et al., 2017). The econometric models included demographic controls (gender, age, educational attainment, income level, work status, marital status, urban residence, and country of residence) and all three social identity measures. The models for the nested decisions (Decision 2 and 3) also included the contribution to the Local account in Decision 1 as a covariate. In this way, the DV measured the propensity to contribute to national and world cooperation net of basic cooperation to the local level.

**Figure 5:**
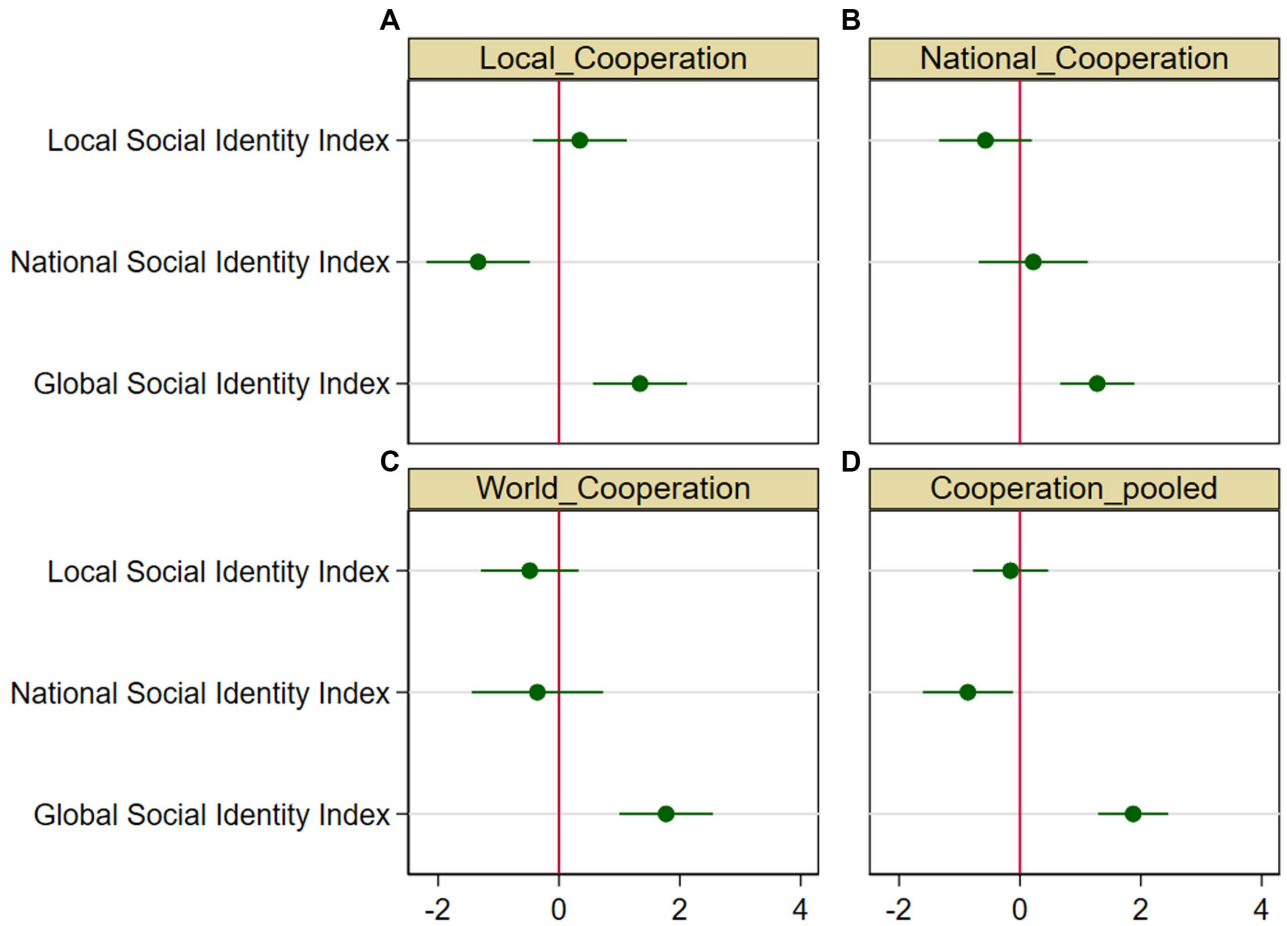
Average marginal effects (AME) and 95% confidence intervals for local, national, and global social identity in regressions. The AME are computed with the delta method as the average of the partial effects on the dependent variable—conditional to lying within the observable range [0, 10]—generated from varying all observations in the regression from a value of 0 to a value of 1 of the independent variable. All other covariates take their actual values. Estimates are taken from the model in column 1 of Appendix: Table A.6 (Local contribution), Appendix: Table A.7 (National contribution), Appendix: Table A.8 (World contribution), and Appendix: Table A.9 (Pooled contributions). See notes to these tables for further details on the econometric models (White, 1980).

The key result emerging from the econometric analysis is that the GSI index was a significant and positive predictor of cooperation at all levels of cooperation, while the LSI index was never significant, and the NSI index was either insignificant or a significant *negative* predictor of local cooperation when other social identity indexes were included in the model. We focus here on the econometric model, including all the three social identity measures together with demographic controls, country dummies, and, for nested decisions, local cooperation in Decision 1. As already reported in Buchan et al. (2011), the GSI index was a significant predictor of cooperation at the world level controlling for both LSI and NSI (*p* < 0.001; Appendix: Table A.8, column 1). The average marginal effect (AME)^1^ of GSI was such that a person who maximally identified with global identity contributed to the World account 1.77 tokens (SD = 0.40; 95% CI [0.99, 2.55]) more than a person who minimally identified with global identity (out of 10 available tokens). Conversely, both NSI (*p* = 0.67; AME = −0.36; SD = 0.55; 95% CI [−1.44, 0.73]) and LSI (*p* = 0.50; AME = −0.48; SD = 0.41; 95% CI [−1.29, 0.32]) had insignificant effects on cooperation at the world level, net of the effect of the two other social identity measures [Figure 5C; Appendix: Table A.8, column 1].

Similar results were attained for cooperation at the national level. The GSI index was significantly and positively correlated with national cooperation controlling for both LSI and NSI (*p* < 0.001). A person who maximally identified with global identity contributed to the National account 1.28 (SD = 0.31; 95% CI [0.67, 1.89]; Appendix: Table A.7, column 1) tokens more than a person who minimally identified with global identity. Conversely, both the NSI index (*p* = 0.63; AME = 0.22; SD = 0.46; 95% CI [−0.68, 1.12]) and the LSI index (*p* = 0.15; AME = −0.57; SD = 0.39; 95% CI [−1.33, 0.20]) had insignificant effects on national cooperation controlling for the two other identity measures (Figure 5B).

Cooperation at the local level was structurally different, because there was only one public good at the local level rather than two nested ones (see section 2.2). However, the results were virtually identical to what was found for national and global cooperation. GSI was significantly and positively associated with local cooperation controlling for both LSI and NSI (*p* = 0.001; Appendix: Table A.6, column 1). A person who maximally identified with global identity contributed to the local public good 1.34 tokens (SD = 0.40; 95% CI [0.56, 2.12]) more than a person who minimally identified with global identity. Conversely, the LSI (*p* = 0.38; AME = 0.34; SD = 0.40; 95% CI [0.56, 2.12]) was insignificant, while the NSI was significant (*p* = 0.002; AME = −1.34; SD = 0.44; 95% CI [−2.20, −0.48]), with a negative sign upon controlling for both LSI and GSI (Figure 5A; Appendix: Table A.6, column 1). These results were robust because of the inclusion of the IGI index and the global awareness index in the model (Appendix: Tables A.6–A.8, column 4), as the coefficient for GSI decreased only marginally after the inclusion of these covariates. This suggests that the correlation between cooperation and GSI was not moderated by either the participation in global networks of interaction or the awareness of global issues.

We examined whether GSI had a different effect on cooperation across the three decisions by pooling the three decisions together and fitting a Tobit panel model, with the participant as the cross-section variable and the level of the decision (local, national, or global) as the “panel” variable of the model. We replicated the previous two models that were used to analyze decisions at a specific level with two modifications: We did not include cooperation at the local level as a control in this case, because this would be collinear with the DV in the first decision. As a consequence, the results from this analysis may not be fully comparable with the results from the previous analyses, and it should also be noted that local cooperation was always a strongly significant predictor of cooperation at the national and global levels (*p* < 0.001, Appendix: Tables A.7, A.8). However, we introduced the level of the decision as a fixed effect in the pooled model. This covariate should control for the different structure of the decisions at the local level vis-à-vis the national and global levels. The results are reported in Appendix: Table A.16 and Figure 5D. Not surprisingly, the main result from the previous analyses was replicated, with GSI being a strongly significant predictor of cooperation, net of the effects of both LSI and NSI (*p* < 0.001). Individuals who maximally identified with global identity contributed on average 1.87 tokens more (SD = 0.30; 95% CI [1.29; 2.45]) than individuals who minimally identified with global identity. Interestingly, we found that national identity had an overall significantly negative coefficient (*p* = 0.024) controlling for both LSI and GSI, with individuals who maximally identified at the national level contributing on average 0.86 tokens (SD = 0.38; 95% CI [−1.61; −0.12]) less than individuals who minimally identified with the national community. The LSI coefficient was not significantly different from zero controlling for both NSI and GSI, the sign being negative (*p* = 0.63; AME = −0.16; SD = 0.32; 95% CI [−0.78; 0.47]).

We then introduced interaction terms between GSI and the level of the decision (Appendix: Table A.16, column 4). Taking GSI at the global level as the omitted category, we found that the coefficient for GSI at the local level was statistically significantly lower than its coefficient at the global level (*p* = 0.012; AME = −0.98; SD = 0.39; 95% CI [−1.74; 0.22]), while there was no statistically significant difference between the GSI coefficient at the national and global level (*p* = 0.61; AME = −0.15; SD = 0.28; 95% CI [−0.70; 0.41]). As noted above, however, the coefficient for GSI at the local level of cooperation was significantly greater than zero. These results indicate that while the GSI exerted its maximal statistical effect at the global and local level, it appears that its correlation with cooperation at the local level was weaker but still significant.

From these analyses, we conclude:

*Result 1a*: Global identification is “inclusive” in character, as the higher the identification with the global collective, the higher the cooperation at any level of interaction—global, national, and local.

*Result 1b*: The correlation between global social identity and cooperation is highest at the global level, statistically insignificantly lower at the national level, and statistically significantly lower at the local level.

*Result 1c*: Identification at the national level tends to be overall negatively correlated with cooperation after aggregating the three decisions. This is particularly the case at the local level.

*Result 1d*: Identification with the local level is overall *in*significantly correlated with cooperation both at the individual level and when pooling the three decisions.

#### 3.2.2. Country-level analysis

Our strategy in selecting countries and the sample size in each country was directed to maximize exposure to globalization across countries rather than identifying significant effects of social identity on cooperation in every single country. All the same, we analyzed the heterogeneity of the relationship between GSI and cooperation across countries. We reproduced the model of Appendix: Tables A.6–A.8, column 1, separately for each country (see Appendix: Tables A.9–A.11). In general, the GSI index coefficients for country-level regressions had the same order of magnitude as those for the aggregate model. Standard errors were, however, on average, 2.5 times higher for country-level regressions than aggregate regressions due to the lower sample size. Therefore, statistical significance was not as strong for country-level regressions as for the aggregate regression. In particular, the GSI coefficient was statistically significantly greater than 0 in regressions with world cooperation as the DV in all countries except for Russia (*p* = 0.79, *N* = 193). It was statistically significantly greater than zero in regressions predicting national cooperation in four countries and outside the region of significance for South Africa (*p* = 0.12, *N* = 121) and the United States (*p* = 0.16, *N* = 163). The significance of the GSI index was somewhat less uniform in cooperation at the local level, as the coefficient resulted as significantly greater than zero in three countries but was insignificant in Iran (*p* = 0.13, *N* = 156), South Africa (*p* = 0.86, *N* = 121), and the United States (*p* = 0.75, *N* = 163).

In order to better appreciate cross-country differences in the relationship between GSI and cooperation, we re-ran the main regressions of Appendix: Tables A.6–A.8, column 1, interacting the GSI index with country dummies. These regressions are reported in Appendix: Table A.12. In this way, we can estimate whether the GSI had significantly different coefficients across countries. The results of all the Wald tests on the null hypothesis of equality of the GSI coefficients across pairs of countries are reported in Appendix: Tables A.13–A.15. This analysis reveals differential patterns in the similarity of the relationship between GSI and cooperation across countries and decisions. The GSI tended to have similar effects across countries in both the local and the national decision. In the local decision, the null was rejected only for two tests (out of the 15 possible) at *p* < 0.05. That was the case for the GSI coefficient being higher in Iran than in the United States (*p* = 0.010) and for the GSI coefficient being higher in Italy than in the United States (*p* = 0.020; Appendix: Table A.13).^2^ Remarkably, no null was rejected in the regression at the national level, denoting uniformity of the relationship between GSI and cooperation at the national level across countries (Appendix: Table A.14). Conversely, the GSI coefficients differed across countries in many instances with respect to global cooperation. It is remarkable that the GSI coefficients were significantly higher in Iran than in any other country. Confirming the lack of a significant relationship between GSI and global cooperation in Russia (see Appendix: Table A.11), the coefficient for Russia was significantly lower than in Argentina (*p* = 0.003) and Italy (*p* < 0.001; see Appendix: Table A.15).

We conclude:

Result 2: The relationship between global social identity and cooperation demonstrates statistical levels of significance in all countries and all decisions, with the notable exceptions of Russia with respect to global cooperation, South Africa and the United States with respect to national cooperation, and Iran, South Africa, and the United States for local cooperation. While the null of equality of coefficients for the GSI across pairs of countries cannot be rejected in any case in the national decision and is rejected in four cases for the local decision, we detect significantly higher coefficients for Iran than any other country in the world decision and significantly lower coefficients for Russia.

#### 3.2.3. Analysis of mediating effect of expectations

In order to better understand the reasons why individuals with stronger global social identification are more cooperative, we examined the mediating effect of expectations. The theoretical model we wanted to test assumes that global social identity increases expectations of others’ cooperation, which in turn induces higher cooperation. We elicited expectation measures by asking the participants to state the total sum of tokens contributed by the three other participants at the local levels and by the 11 other participants at the national and global levels (see Supplementary Methods: Section SM.3). The highest possible number of tokens available for contribution (30 for the local level, 110 for the national and world levels) was mentioned to the participants in their answer sheets. We asked the total number of tokens rather than the average number of tokens, because we expected the former to be of easier comprehension than the latter for our adult sample. All expectation measures were normalized to the [0,10] interval.

We performed a Sobel–Goodmann (SG) test (Sobel, 1982) on the above hypothesis (see Appendix: Section A.4 for a description of the mediation analysis). In section 3.2.1, we already mentioned that the coefficient for the GSI index was strongly significantly different from zero in all decisions; hence, the total effect was significant (Appendix: Tables A.6–A.8, column 1). Moreover, at all three levels, the coefficient for the GSI index was significantly greater than zero when the DV was the expectation of contribution at the corresponding level. This confirms that global identification was significantly correlated with expectations, while this was not the case for either the LSI or the NSI indexes (Appendix: Tables A.6–A.8, column 2). Moreover, expectations were significant predictors of cooperation at all levels (*p* < 0.001 in all three models; Appendix: Tables A.6–A.8, column 3). Table 1 confirms that the indirect effect of GSI was significant at all three levels and that the proportion of the total effect that is mediated by expectations is considerable (44% for local cooperation, 25% for national cooperation, and 40% for global cooperation), even as the direct effect of global social identity remained strongly significantly different from zero in all cases. We conclude:

Result 3: Individuals with high global social identity form significantly higher expectations of cooperation at all three levels of their counterparts’ cooperation and cooperate more with others. This indirect effect of global social identity on cooperation is sizable and significant at all three levels, but so is its direct effect.

**Table 1:** Sobel–Goodman mediation analysis.

Level	Statistic	Total effect	Indirect effect	Direct effect
Local (Decision 1)	Coefficient	1.33	0.59	0.74
	Bootstrap std. err.	0.38	0.19	0.31
	*p* value	<0.001	0.002	0.017
	99% confidence interval	[0.35, 2.30]	[0.010,1.07]	[−0.061, 1.54]
	Proportion of total effect that is mediated	0.44		
National (Decision 2)	Coefficient	1.21	0.30	0.91
	Bootstrap std. err.	0.31	0.12	0.28
	*p* value	<0.001	0.0014	0.001
	99% confidence interval	[0.42, 2.00]	[−0.014,0.61]	[0.18, 1.64]
	Proportion of total effect that is mediated	0.25		
World (Decision 3)	Coefficient	1.57	0.63	0.94
	Bootstrap std. err.	0.36	0.17	0.32
	*p* value	<0.001	<0.001	0.003
	99% confidence interval	[0.65, 2.49]	[0.19,1.06]	[0.12, 1.76]
	Proportion of total effect that is mediated	0.40		

#### 3.2.4. Analysis of optimism of expectations

The foregoing analysis has demonstrated the importance of expectations in affecting cooperation levels, especially for individuals with high levels of global social identity. We now analyze whether such expectations were misplaced (see Introduction). We define the variable *Optimism* (*O*) as the difference between a participant’s expectation of contribution at a certain level and the actual population mean contribution at that level.^3^

For instance, *O* at the local level is the difference between how much an individual expects others to contribute at the local level in Decision 1 and the actual mean contribution at the local level in Decision 1. Clearly, *O* > 0 denotes people tending to be optimistic of others’ contributions, while *O* < 0 denotes pessimism. Over the whole sample, pessimism had a slight predominance, with 52.4% of participants having on average a negative *O*. A Wilcoxon sign-rank test failed to reject the hypothesis that the distribution of *O* was symmetrically distributed around zero (*z* = −0.81, *p* = 0.42, *N* = 1,108). Cross-country differences in optimism are analyzed in the Appendix: Section A.5 (see also Appendix: Figures A.2–A.3).

We fitted a series of OLS regressions where the DV was Optimism at the local level (Decision 1), national level (Decision 2), and global level (Decision 3) and the mean over these three levels (Appendix: Table A.17). Individuals with stronger global social identity were significantly more optimistic than individuals with weaker global social identity at the local (*p* = 0.005), national (*p* = 0.001), and world level (*p* < 0.001); thus, they resulted as significantly more optimistic than others across the three decisions (*p* < 0.001). Conversely, Optimism did not covary with either LSI or NSI. Among the demographic characteristics, individuals with intermediate (*p* = 0.045) or high levels of education (*p* = 0.046) tended to be overall more optimistic across the three decisions than individuals with low educational attainment (Appendix: Table A.17, column 4), particularly so at the local and global levels.

#### 3.2.5. Analysis of accuracy of expectations

The fact that individuals with strong global social identity were significantly more optimistic than others does not entail that they were more inaccurate in their predictions. Pessimistic individuals may commit an even larger error than optimistic individuals by underestimating others’ contributions more than optimists overestimate others’ contributions. In order to analyze the accuracy of the prediction, we need to consider the forecast error (*FE*), namely, the absolute level of the distance between one’s expectation and the actual level of cooperation.^4^

Since it abstracts away from the sign of the error, the *FE* permits a direct comparison of the error in prediction by optimists and pessimists. The closer the *FE* is to zero, the higher the accuracy of the prediction.

**Figure 6:**
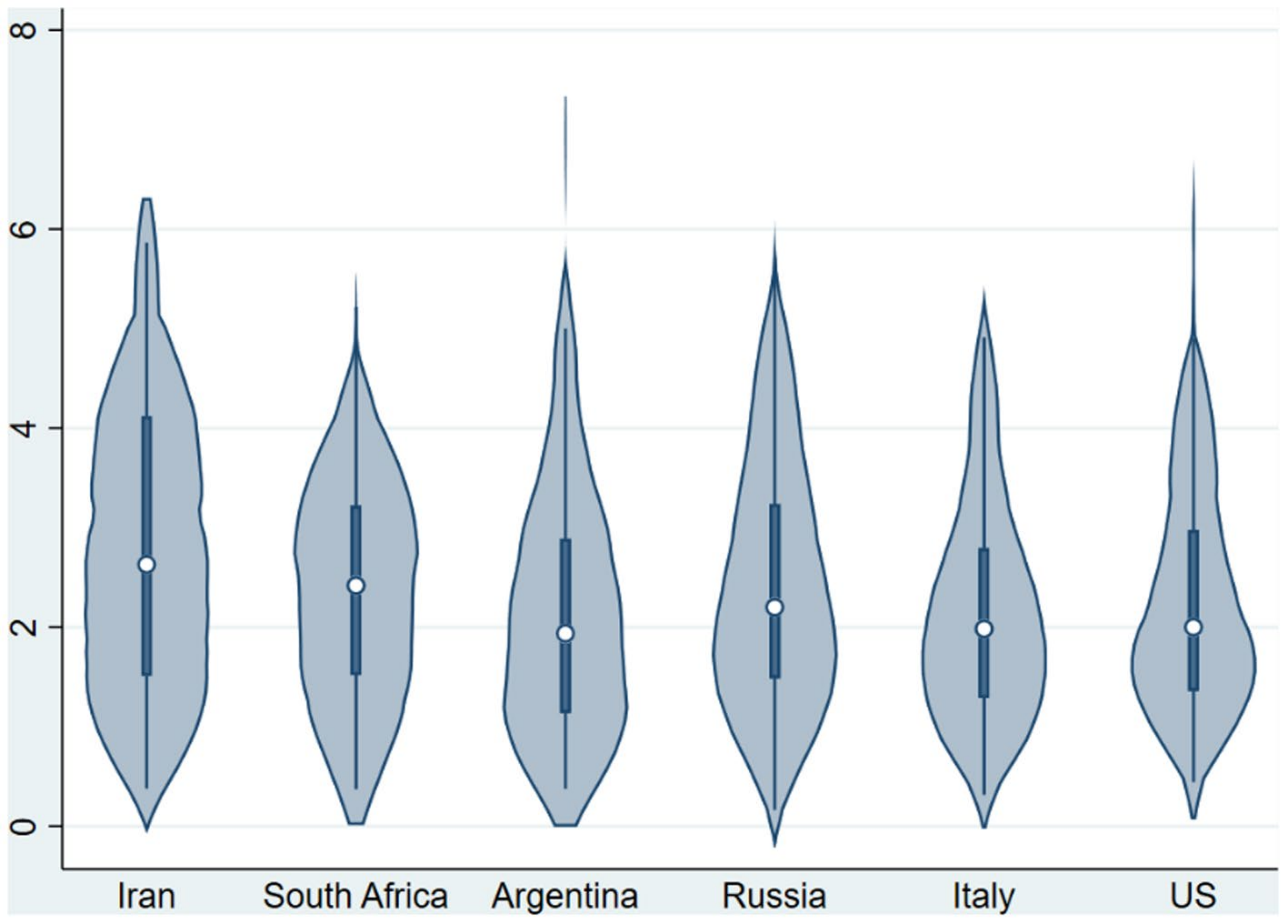
Distribution of forecast error per country (mean over Local 1, National 2, and World 3).

Figure 6 plots the distribution of the mean *FE* over contributions at the local (in decision 1), national, and global level across countries. The forecast error was substantial, as the mean of *FE* was 2.36 tokens (out of 10). The lower bound of a 99% confidence interval with bootstrapped s.e. (5,000 repetitions) for the mean of *FE* is well above zero ([2.27, 2.45]). The null hypothesis that the observations from individual countries come from the same distribution was rejected [chi^2^(5) = 29.05, *p* = 0.0001, *N* = 1,108]. We report a descriptive analysis of country-level differences in Appendix: Section A.6.

We fitted OLS regressions with the *FE* as the DV and the same set of covariates used above (Appendix: Table A.18). We found that the participants with strong identification at the global level were no more inaccurate than others at both the local (*p* = 0.70) and national (*p* = 0.24) levels, while they were significantly more inaccurate than others (*p* < 0.001) in predicting cooperation at the world level. In this case, an individual with a maximal GSI score would commit an FE 0.80 tokens higher than an individual with a minimal GSI score. Therefore, only in the third decision can we say that individuals with a high GSI were “excessively” optimistic in estimating others’ contributions. That was not the case for cooperation at the local and national levels. Considering the mean over the three levels, the coefficient for the GSI index was significantly greater than zero (*p* = 0.023).

We conclude:

Result 4: Individuals with stronger global social identity were significantly more optimistic than others regarding their counterparts’ cooperation at any level of interaction. Nonetheless, their predictions turned out to be significantly more inaccurate than others’ only at the world level, but not at the local or national levels. In other words, their optimism was “excessive” only at the global level, but not at the local or national levels.

## 4. Discussion

As in every study attempting inference from relatively limited samples, our study is subject to a series of limitations both in terms of its internal and external validity. As for its internal validity, some may question whether the participants in our study achieved a full comprehension of the task. We believe we applied best-practice techniques in our study to ensure their full comprehension. The instructions used pictorial illustrations of the interaction that we deemed suitable for adults possibly lacking computational abilities (see Appendix: Supplementary Methods SM.3). We also administered a set of comprehension questions before the first and the second decision. In case of failure in answering such questions correctly, the researchers would explain the interaction again until the questions were answered satisfactorily. Internal validity was also ensured by the standardization of the experimental protocol and by compliance with best-practice techniques to ensure comparability across countries (see Supplementary Methods SM.2). In particular, each lead researcher observed each other before running the experimental sessions or, in the case of Iran, was instructed by one of the lead researchers. Instructions were backtranslated into English to ensure homogeneity in the language being used. The value of the experimental tokens was adjusted to reflect differences in purchasing power across countries.

An aspect of our study that warrants further investigation is the specific language that was used to measure global social identity. The categories used to identify the three levels of social identification—“Your local community” for the local level; “Your country” for the national level; “The world as a whole” for the global level (Appendix: SM4 Research Questionnaire, questions 21–23)—were not, and could not have been, fully homogenous because of the intrinsic differences between the three entities. The use of the term “world as a whole” was derived from the pioneering work by Robertson (1992), who considered the “consciousness of the world as a whole” as a defining aspect of globalization. We could not verify whether the participants construed this wording in terms of “people all over the world” or “all humanity” or in the even broader sense of encompassing all animal and non-animal species living on planet Earth.^5^ Although these concerns about the robustness of the construct are legitimate, we believe it is reassuring to observe strong similarities in research using this construct and the IWAH construct (see Introduction).

Another issue concerns the external validity. Experimental techniques have been criticized for the possibility that behavior in the lab is driven by experimenter demand effects and social desirability bias. That is, participants would bias their behavior in the direction of what they perceived as being the behavior desired by the researcher or the socially approved behavior (Levitt and List, 2007; Zizzo, 2010). Even if the evidence supports the idea that experimenter demand effects are sizable and that individuals tend to behave more prosocially when under the researcher’s scrutiny (Levitt and List, 2007), this bias normally only affects the baseline level of cooperation and not the treatment effects. The systematic study conducted by Snowberg and Yariv (2021) supports the idea that treatment effects tend to be of the same magnitude across different samples even if the baseline levels may differ. They reached this conclusion by running the same experimental games with university students—either self-selected or not—and with nationally representative adult samples of the US population. One may argue that if social desirability does not apply uniformly across treatments, the treatment effects may be distorted. Nevertheless, this concern does not seem to be the case in our setting. Even assuming that the participants increased their cooperation levels due to an experimenter demand effect, it is not clear why they should have done so differently across the different levels of cooperation objects of our experiment.

The issue of the external validity of social preference games has also been analyzed by Galizzi and Navarro-Martinez (2019). Their meta-analysis of existing experimental studies on prosocial behavior reveals that *“39.7% of the reported lab-field correlations and 37.5% of the reported lab-field regressions find a statistically significant association between games and field behaviors. The overall average lab-field correlation reported is 0.14, and the overall correlation in the papers that report significant correlations is 0.27*.*”* Field behaviors include (mostly) self-reported prosocial behavior elicited through surveys and real-life prosocial behavior observed by researchers. For instance, Rustagi et al. (2010) find that experimental measures of conditional cooperation among 49 forest user groups (*n* = 679) in Ethiopia significantly correlate with more successful forest commons management. Fehr and Leibbrandt (2011) find that laboratory measures of cooperation are significant predictors of exploitation of fisheries—a typical common pool resources interaction—among rural fishing communities in Brazil (*n* = 121). Grimalda et al. (2018) find that individuals who cooperated in a collective risk social dilemma experiment were more likely to undertake environmentally sustainable behavior in real life, such as buying environmentally friendly goods, saving water, participating in ecological movements, and recycling (*n* = 678).

This evidence suggests that prosocial behavior measured in experiments is, overall, a significant predictor of prosocial behavior in real life, although the correlation is not always strong or significant (see also the experiment by Galizzi and Navarro-Martinez, 2019). Failure to observe a stronger and more consistent correlation between experimental behavior and real-life behavior may be partly due to the intrinsic inconsistency of human behavior over time. It has been observed that the same individual may behave differently in similar situations, possibly for moral licensing—that is, the tendency to indulge in more opportunistic behavior after having performed moral deeds (Merritt et al., 2010)—or because of the phenomenon of preference reversal in dynamic choices involving social preferences under uncertainty (Andreoni et al., 2020).

Although our design was not meant to identify statistically significant relationships at the country level (see section 3.2.2), the country-level analysis revealed interesting insights into the extent to which GSI correlated with cooperation differently across countries. While the country-level specification reveals the lack of significance of GSI in some countries (see section 3.2.2), the analysis of the interaction between GSI and country dummies reveals that GSI tended to have uniform effects across countries in the national decision. In the local decision, we found limited evidence for differential effects of GSI across countries, with Iran and Italy recording significant larger effects than the United States. As for the global decision, it is noteworthy that the GSI coefficient is significantly higher in Iran than in any other country, while it is low and not significantly different from zero in Russia. This suggests that the effect of global social identification on global cooperation tends to be overall higher in countries with *lower* levels of globalization, as also investigated in Grimalda (2015). Finally, we point out that these cross-country comparisons (Appendix: Section A.2) should be treated with caution, since we did not test measurement invariance, which is recommended before concluding such differences (e.g., Hamer et al., 2021).

## 5. Conclusion

The results from the present study demonstrate that global social identity is inclusive in character. Not only do globally identifying individuals cooperate more than others at the global level, but they also cooperate more than others when involved in local and national group interactions. The effect of global identification on cooperation at all levels is distinct from both participation in global networks and awareness of global humanitarian concerns, as demonstrated in Buchan et al. (2011) for global cooperation. Furthermore, when we contrasted the effect of global identity with social identity that is primarily local and national in nature, the latter failed to demonstrate any significant independent effect on cooperation at any level of interaction. In other words, global social identification appears to be the only form of social identity that is significantly associated with cooperation at all levels. Even if the strength of the relationship between GSI and cooperation is higher at the global level than at the local level, it remains strongly significant and sizable at the local level too.

In their review of research on global human identity, McFarland et al. (2019) state that for those with strong global identity, “group behavior ascends from parochial interests (e.g., ‘American first’) to solidarity and care for all humans” (p. 144). The results from this study complement this view and provide evidence that concern for global welfare *does not* come at the expense of more parochial (local or national) interests. On the contrary, individuals with strong identification with the global collective cooperate more than others at both the local and national level. This is the case in non-nested social dilemmas at the local level and in nested social dilemmas at the national level.

The inclusive nature of global social identity, therefore, entails that those who score high on identification with the global community are also willing to benefit collectives at other levels. This result is in contrast with the view generally held in political science that global and national identities are substitutes of each other (see section 1). It is also consistent with the view that prosociality is universal rather than parochial (see section 1), although this is the case specifically for individuals with high GSI. On this point, we also note that we did not find strong evidence for an ingroup bias, which is measured by the difference in contributions to the national vis-à-vis the global account. A sign-rank Mann–Whitney–Wilcoxon test failed to reject the hypothesis of an ingroup bias in the aggregate of our data (*p* = 0.54; *N* = 1,112) and only rejected the null for Argentina (*z* = 2.66, *p* = 0.0079; *N* = 201) and South Africa (*z* = 1.92, *p* = 0.055; *N* = 159) for individual countries.^6^

However, it is surprising that their level of prosociality toward local and national groups *exceeds* that of those who identify strongly only at the more local levels, net of the identification at the other levels. National social identity does not predict contributions to the national pot as strongly as does global social identity (and similarly for local identity). This suggests that those high in global social identification are not only more inclusive in their social ingroup identity but also more concerned about collective (vs. individual) welfare in general.

We also investigated whether these patterns of cooperation are due to misperceptions of others’ cooperation, as suggested in the literature on prosociality. We found that individuals with stronger global social identity are more optimistic about others’ cooperation at all levels of interaction. When we analyzed the accuracy of their prediction, though, we found that they are “excessively” optimistic only at the global level. When individuals with strong identification with the global collective interact at the local and national levels, they are no more inaccurate than others with weak identification with the global collective. This result suggests that individuals with high global social identity may be spurred by partially different motivations when interacting at the global level as opposed to lower levels of inclusiveness. Interaction at the local and national levels for them appears closer to a model of reciprocity (Fischbacher et al., 2001), where an individual contributes in line with the cooperation expected from others. Interaction at the global level seems, instead, to demonstrate a revealed preference for the idea of solidarity and care for all humans. Overall, these results point to distinctively different patterns of behavior by individuals with a stronger identification with global collectives than others.

Given the converging evidence on the clearly beneficial patterns of behavior displayed by individuals with a high global social identity, we believe that a promising avenue of research is to understand whether higher identification with the global community may be somehow instilled into individuals, possibly through appropriate educational programs, or whether the personality traits referring to global identity are non-malleable to external intervention. The research of our research group suggests that simple “nudging” to the global dimension in the context of COVID-19 does not induce greater donations to the local, national, or global levels (Grimalda et al., 2021), a result echoing that of Sparkman et al. (2022). This does not necessarily mean that this endeavor is bound to fail, but rather, as argued by Ostrom (2000) in the context of programs to increase social capital, that one has to try harder and for a protracted period of time.

## Data availability statement

Publicly available datasets were analyzed in this study. This data can be found at: https://osf.io/8trzj/.

## Ethics statement

The studies involving human participants were reviewed and approved by Ethics committee at Darla Moore School of Business, Sonoco International Business Department, University of South Carolina, 1,014 Greene Street, 461G, Columbia, SC 29208. Written informed consent for participation was not required for this study in accordance with the national legislation and the institutional requirements.

## Author contributions

GG, NB, and MB designed research, conducted fieldwork, and wrote the paper. GG conducted statistical analyses. All authors contributed to the article and approved the submitted version.

## Funding

This work was supported by the National Science Foundation (Grants 0652277 and 0652310), the Center for the Study of Globalization and Regionalization (CSGR) at the University of Warwick, the Center for International Business Education and Research (CIBER) at the University of South Carolina, the Laboratory for Research in Experimental Economic (LINEEX) at the University of Valencia, the Spanish Ministry of Science and Education (Grants SEJ2007-66581 and ECO2008–04784), the Canadian Social Sciences and Humanities Research Council, and the Guanghua School of Management, University of Peking. Organizational support from the Center for Research and Education in Economic Development (CIDED) in Argentina and Econometica in Italy is also gratefully acknowledged.

## Conflict of interest

The authors declare that the research was conducted in the absence of any commercial or financial relationships that could be construed as a potential conflict of interest.

## Publisher’s note

All claims expressed in this article are solely those of the authors and do not necessarily represent those of their affiliated organizations, or those of the publisher, the editors and the reviewers. Any product that may be evaluated in this article, or claim that may be made by its manufacturer, is not guaranteed or endorsed by the publisher.

## Footnotes

^1^See footnote 5 for definition of the AME.

^2^Two of such tests were rejected at *p* < 0.10. See Appendix: Table A.13.

^3^In formulas, $O_{i}^{L_{j}}=E_{i}\left(C^{L_{j}}\right)-\bar{C^{L_{j}}}$ where $E_{i}\left(C^{L_{j}}\right)$ is the expectation of individual *i* over contribution at level *L* in decision *j*, where L = {local, national, global}, and j = {1, 2, 3]; $\bar{C^{L_{j}}}$ is the population mean contribution at level *L* in decision *j*.

^4^In formulas, $FE_{i}^{L_{j}}=\left|E_{i}\left(C^{L_{j}}\right)-\bar{C^{L_{j}}}\right|$ . See footnote 3.

^5^See Carmona et al. (2020) for an inquiry into differences in construals of various labels identifying all-inclusive identities.

^6^The other tests failed to reject the null of absence of ingroup bias in Iran (*z* = −1.64, *p* = 0.1006; *N* = 169), Russia (*z* = −1.41, *p* = 0.16, *n* = 207), Italy (*z* = 0.50, *p* = 0.61, *n* = 205), and the United States (*z* = −0.55, *p* = 0.58, *n* = 171). It is worth nothing that in Iran and Russia, participants gave on average more at the world than the national level.
