# Supplementary material for: Global social identity predicts cooperation at local, national, and global levels: Results from international experiments
Source: Front Psychol. 2023 Jun 30;14:1008567. doi: 10.3389/fpsyg.2023.1008567 (PMC10349348; doi:10.3389/fpsyg.2023.1008567)
Supplement: Supplementary file 1 [file Data_Sheet_1.pdf]

Global social identity predicts cooperation at local, national, and global levels: Results from international experiments

Appendix

Note: In order to access any of the contents below, place your mouse over the desired content and press Ctrl and left-hand click.

Table of Contents

Section A.1: Notes on the Country-level and Individual-level Globalization Indexes...... 3

Section A.2: Supplementary analyses of social identity scores.. 5

Section A.3: Notes on Tobit regression... 6

Section A.4: Notes on mediation analysis 7

Section A.5: Cross-country differences in optimism... 8

Section A.6: Cross-country differences in the Forecast Error 9

Supplementary Tables.. 10

Table A.1: Background data on the Country-level Globalization Index 10

Table A.2: Sample descriptive statistics by country.. 11

Table A.3: Summary of parameters in experimental decisions 12

Table A.4: Cronbach's alpha of social identity measures.. 13

Table A.5: Correlation between cooperation and social identity measures..................... 13

Table A.6: Local level analysis-All countries.. 17

Table A.7: National level analysis-All countries... 19

Table A.8: World level analysis-All countries 21

Table A.9: Local level analysis-Individual countries 22

Table A.10: National level analysis-Individual countries........ 23

Table A.11: World level analysis-Individual countries 24

Table A.12: Interaction between GSI and countries 25

Table A.13: Results of Wald tests on null hypothesis that GSI coefficients differ in pairs of countries – Local level 26

Table A.14: Results of Wald tests on null hypothesis that GSI coefficients differ in pairs of countries – National level 27

Table A.15: Results of Wald tests on null hypothesis that GSI coefficients differ in pairs of countries – Global level 27

Table A.16: Pooled regression 28

Table A.17: Analysis of optimism 30

Table A.18: Analysis of Forecast error 31

Supplementary Figures 32

Figure A.1: Description of mediation effect 32

Figure A.2: Distribution of Optimism per country 33

Figure A.3: Mean optimism by country and decision........ 34

Figure A.4: Mean forecast error by country and decision 34

Supplementary Methods 35

SM.1: Choice of Locations and Recruitment Procedures 35

SM.2: Summary of Experiment Protocol........ 36

SM.3: Experiment Script (Version used in a US location) 39

SM.4: Research questionnaire 65

SM.5: Further notes on construction of the IGI index 82

Section A.1: Notes on the Country-level and Individual-level Globalization Indexes

The Country-level Globalization Index (CGI)

The Country-level Globalization Index (CGI) developed by the Centre for the Study of Globalisation and Regionalisation (CSGR) (1) gauges the economic, social and political dimensions of globalization for a sample of 104 countries and combines them into an overall globalization index for each country for the years 1982-2004. The list of variables on which the index is computed is reported in Table A.1-Panel A. Scores for each of the economic, social, and political domains are computed for each country. These are then combined to yield the overall Country-Level Globalization Index (CGI). Such scores are reported in Table A.1-Panel B, along with the ordinal ranking of a country within the sample. For comparative purposes, we have also included the scores for the country at the top (Singapore) and at the bottom (Samoa) of the overall ranking. Our sample of countries, though numerically limited, covers a broad range of the globalization spectrum - although admittedly countries at the bottom end of the scale are under-represented.

The Individual-level Globalization Index (IGI)

The Individual-level globalization index (IGI) was constructed to be analogous to both the CSGR and the Foreign Policy/A.T. Kerney (2005) indexes of country-level globalization, although some importance differences between the individual and country-level constructs remain. The content of the questionnaire is specified by the theoretical conceptualization of globalization outlined in the paper. The IGI measures the extent to which individuals interact with and potentially become interdependent with distal others

in economic, social and cultural interactions. The 33 items which form the IGI are reported in Supplementary Methods: SM.5.

Most questions on the IGI are Likert scaled, with the lowest category denoting a lack of ownership or access of a particular medium of connection and highest category denoting the highest frequency of use or interaction (see e.g. section SM.4, Question 3b). Some questions inquire as to the scope of activity; for example, whether someone uses their mobile phone to contact people in their locality, other parts of their country, or other countries (see e.g. Section SM.4, Question 2b). Finally, other questions are “yes” / “no” in response format; for example, whether someone works for a multinational company; these questions purely gauge participation or non-participation in globalization (see e.g. Section SM.4, Question 9).

The scores to each question have been reverse-scored when necessary, and normalized to the [0,1] interval, such that a score of 0 always corresponds to the lowest possible occurrence of an event or circumstance - e.g. a lack of access to an international news source, and a score of 1 is associated with the maximum possible occurrence - e.g. highest possible frequency in watching or listening to an international news source. The normalized scores have then been summed up and divided by the number of the questions answered by the individual. As for the multiple choice questions asking the area (local, national, international) in which a subject carries out a certain activity, we assigned a lexicographic score reflecting the broadest area within which the subject has interactions. That is, scores of 1/0.5/0.25 were assigned if the subject answered she has international/national/local interactions, respectively, and a score of 0 if the subject has no such interaction.

Section A.2: Supplementary analyses of social identity scores

We report the Cronbach's alphas for the three social identity scales in Table A.4. In all cases except Russia, the alphas are above 0.7 suggesting reliability of the indexes. In Russia, the reliability of the indexes is questionable, as the alphas are between 0.55 and 0.65. A principal component analysis for Russia reveals that the only eigenvector greater than 1 is that associated with the first component (1.77 for the local; 1.63 for the national; 1.75 for the global social identity index). This suggests uni-dimensionality of the indexes even for Russia, although the proportion of variance explained by the first component is less than 60%. Among the three items, it is the attachment dimension that is the least correlated with the other two.

According to non-parametric Wilcoxon sign-rank tests for matched pairs, the difference between the LSI and the NSI is not significant in any country except for the Russian Federation ($p=0.0003$, $N=205$), where participants tend to report higher LSI than NSI, although the effect size is small (Cohen's $d=0.13$) – and Italy ($p= 0.0004$, $N=203$) where on the contrary participants report lower LSI than NSI with a small to medium effect size ($d= -0.26$). The result in Russia may be driven by the ethnic diversity of the sample, as people with ethnicity different from Russian identify more with local than national identity. Merging all observations together, there was no statistically significant difference between the distributions of LSI and NSI ($p=0.20$, $N=1093$; $d= -0.08$). On the contrary, the difference in the means of LSI and GSI had a medium effect size ($d=0.59$) and the test of equality of distributions was soundly rejected ($p<0.0001$, $N=1086$). The same occurred for the difference in distributions between the NSI and GSI ($p<0.0001$, $N=1086$), with an even larger effect size ($d=0.68$). It is noteworthy that for every country

except the US the null hypothesis of equality of distributions between the LSI and GSI was always rejected at p<0.0001 with effect sizes ranging from small to medium in Italy (d=0.34) to large in Iran (d= 1.29). Likewise, the null of equality of distributions between the NSI and GSI was always rejected at p<0.0001 in all countries except the US, with effect sizes ranging from medium in Russia (d= 0.49) to large in Iran (d= 1.29). In the US, the null of equality of distributions for LSI and GSI was not rejected at conventional levels of significance (p=0.069; N=170, exact test; d= 0.15). The null of equality of distributions for NSI and GSI was instead rejected in the US as well (p= 0.0025; N=170, exact test) but with only a small effect size (d=0.24). Overall, this analysis suggests that the US sample had relatively higher GSI compared to the other two social identity indexes than other countries. In Iran, conversely, the GSI was relatively lower than the other two social identity indexes than in the other countries.

Virtually identical results obtain applying ANOVA tests on the difference in means (not reported; see Supplementary Materials: Analyses codes and statistical analysis output). Since measurement invariance hardly ever reaches the level allowing for cross-country comparisons of means, results are to be taken with caution (Hamer et al., 2021).

Section A.3: Notes on Tobit regression

A Tobit estimator uses maximum likelihood estimation methods under the assumption that a latent partially observable variable generated the data. The relationship between the latent variable and the observed variables is described by the following mapping:

$$\boldsymbol{C_i^* = Z_i'\varphi + \theta_i} \qquad (1)$$

$$\boldsymbol{C_i} = \begin{cases} \mathbf{10} & \boldsymbol{if \quad C_i^* \geq 10} \\ \boldsymbol{C_i^*} & \boldsymbol{if\ 0 < C_i^* < 10} \\ \mathbf{0} & \boldsymbol{if\ C_i^* \leq 0} \end{cases} \qquad (2)$$

In our case, the latent variable is an individual's propensity to cooperate. Theoretically, an individual may want to contribute less than 0 tokens or more than the 10 tokens available for contribution. Hence, while we can observe directly the latent variable when its manifestation is in the [0, 10] range, we will only observe a choice of either 0 or 10 when the latent variable is less than 0 or greater than 10. The tobit estimator assumes a linear model for the latent variable, where Z_i' is the set of the independent variable, φ are the estimated parameters, and θ_i is a normally distributed error term. The estimator corrects the likelihood function introducing the estimation of the probability that $\boldsymbol{C_i^*} \geq 1\mathbf{0}$ and that $\boldsymbol{C_i^*} \leq 0$. The estimated φ is then a combination of the marginal effect of a change in an independent variable onto the latent variable in the region $0 < \boldsymbol{C_i^*} < 1\mathbf{0}$ and of the marginal effect on the probability that $\boldsymbol{C_i^*} \geq 1\mathbf{0}$ and that $\boldsymbol{C_i^*} \leq 0$.

Section A.4: Notes on mediation analysis

The Sobel Goodman test decomposes the Total effect exerted by the independent variable onto the DV (path c in Appendix: Figure A.1) into a Direct effect and an Indirect effect. The Direct effect is given by the effect of the Independent variable onto the DV when a Mediating variable is included in the model (path *c'* in Figure A.1). The Indirect effect is the effect exerted by the Independent variable onto the DV through the Mediating variable (path ab in Figure A.1). The Indirect effect is the difference between the Total effect and the Direct effect, which is equal to the product of the coefficients *a* and *b* in the regression including both the independent variable and the mediating variables.

Section A.5: Cross-country differences in optimism

Optimism is the difference between a participant's expectation of contribution at a certain level and the actual population mean contribution at that level (see section 3.2.4 and footnote 3 in the main paper). A Kruskall Wallis test rejected the hypothesis that the observations of $OPT_i^{L_j}$ (defined in equation (1)) in the six countries were generated by the same distribution (chi2(5) =77.97; p=0.0001, N=1108). In fact, 68.2% of Iranian participants and 69.9% of South African participants had negative $\overline{OPT_i^{L_j}}$, the null hypothesis of symmetry of distribution around zero being rejected both in Iran (z = -3.88, p=0.0001, N=179) and in South Africa (z = -5.063, p<0.0001, N=153). In Argentina and Russia, optimism prevailed, the hypothesis of symmetry of the distribution being rejected for both Argentina (z = 3.58, p=0.0003, N=199) and Russia (z = 3.44, p=0.0006, N=204). Finally, optimism prevailed in Italy while pessimism was marginally dominant in the US, but the hypothesis of symmetry of the distribution around the zero was not rejected in either Italy (z = 1.28, p=0.20, N=202) or the US (z = -1.01, p=0.31, N=171). Figure A.3 plots mean optimism by country and decision. It is striking that pessimism prevails in the local decision in every country except for Argentina. Optimism is prevalent in Argentina and Russia for national contributions. In the world decision, pessimism is highest in Iran and South Africa while optimism prevails in the Russian Federation and the US.

Section A.6: Cross-country differences in the Forecast Error

Figure A.4 plots mean accuracy by country and decision (see section 3.2.5 in the main paper for the definition). Overall, accuracy is highest in the local decision (mean=2.14, s.d.=1.50), intermediate for national contribution (mean=2.30, s.d.=1.61) and lowest for world contribution (mean=2.62, s.d.=1.65). Iran is the country with the lowest accuracy in all three decisions. Mann-Wilcoxon non-parametric tests (see output from analysis reproduction codes) reject the null hypothesis of equality of distributions in all pairwise tests involving Iran and all other countries ($p<0.026$ or less) except for the US ($p=0.13$, $N=350$). In the national decision, accuracy in Iran was not significantly different from accuracy in any other country except for Italy ($p=0.0088$, $N=378$). Finally, in the world decision accuracy in Iran was significantly lower than in any other country ($p=0.040$ or less).

US participants had the second highest forecast error in the local decision. The null of equality of distribution was rejected in pairwise tests with Argentina ($p<0.0001$, $N=368$), the Russian Federation ($p=0.028$, $N=368$) and Italy ($p=0.0075$, $N=373$).

Argentineans had the lowest forecast error in the local decision, and the null of equality of distribution was rejected in all pairwise tests with any country ($p<0.036$ or less) except for Russia ($p=0.061$, $N=394$).

Italians were the most accurate with regard to national cooperation, but the null of equality of distributions was rejected only in the pairwise test with Russia ($p= 0.0055$, $N=399$) and Iran ($p=0.0088$, $N=378$).

As for World cooperation, the null of equality of distributions was never rejected in any pair of countries not involving Iran.

Supplementary Tables

Table A.1: Background data on the Country-level Globalization Index

Sub-Index	Variables
Economic globalization	Trade
	Foreign Direct Investment
	Portfolio Investment
	Non-residents income and income of residents working abroad
Social globalization (People)	Stock of Foreign Population
	Flow of Foreign Population
	Worker Remittances
	Tourists
Social globalization (Ideas)	Phone calls
	Internet users
	Films
	Books and newspapers
	Mail
Political globalization	Embassies
	UN Missions
	Organisations

(A) List of variables included in the Country-level Globalization Index (CGI)

Country	Economic Globalization Score	Social Globalization Score	Political Globalization Score	CGI	Ordinal ranking
Singapore	*0.43*	*0.99*	*0.26*	*0.95*	*1*
USA	0.13	0.55	0.87	0.87	5
ITALY	0.13	0.40	0.71	0.67	13
RUSSIA	0.16	0.12	0.85	0.60	19
ARGENTINA	0.12	0.12	0.55	0.38	37
SOUTH AFRICA	0.14	0.07	0.51	0.34	48
IRAN	0.13	0.02	0.35	0.20	74
Samoa	*0.09*	*0.05*	*0.09*	*0.04*	*104*

(B) Country scores for Country-Level Globalization Index

Note: Panel B reports scores for each sub-index of the Country-Level Globalization Index (CGI), the aggregate level of CGI and the ordinal ranking for the six countries included in our study and for the country at the top (Singapore) and at the bottom (Samoa) of the aggregate CGI at the time the research was run.

Table A.2: Sample descriptive statistics by country

Country/ Statistics	Obs.	Income [1=Highest Decile in a country's income distribution]	Education [1=Higest educational attainment]	Age	Gender [1=All females]	IGI [1=Most globalized]	CGI [1=Most globalized]
IRAN	159						
Mean		0.308	0.428	38.363	0.503	0.286	0.20
St. Dev.		0.23	0.24	16.27	0.50	0.14	
Median		0.333	0.6	37		0.282	
SOUTH AFRICA	159						
Mean		0.576	0.347	36.979	0.648	0.410	0.34
St. Dev.		0.31	0.21	16.18	0.48	0.17	
Median		0.556	0.2	31		0.404	
ARGENTINA	195						
Mean		0.700	0.322	39.416	0.571	0.397	0.38
St. Dev.		0.27	0.21	11.98	0.50	0.13	
Median		0.778	0.2	39		0.411	
RUSSIA	196						
Mean		0.570	0.437	40.709	0.565	0.390	0.60
St. Dev.		0.30	0.17	14.53	0.50	0.12	
Median		0.556	0.4	42		0.375	
ITALY	187						
Mean		0.499	0.419	40.390	0.520	0.417	0.67
St. Dev.		0.29	0.14	14.30	0.50	0.12	0.00
Median		0.444	0.4	40		0.419	
USA	164						
Mean		0.362	0.402	40.586	0.465	0.406	0.87
St. Dev.		0.25	0.24	15.71	0.50	0.11	
Median		0.333	0.4	40		0.394	
Total	1029						
Mean		0.507	0.394	39.532	0.543	0.385	0.52
St. Dev.		0.31	0.21	14.80	0.50	0.14	0.22
Median		0.444	0.4	39		0.383	

Notes: All the variables apart from Age are scaled on the [0 , 1] interval.

Income denotes the income decile to which a subject responded s/he belongs within his/her country income distribution. It is therefore to be understood as a country-specific measure. See Section SM.4, Question 38.

Education is the highest level of education attained by an individual. See Section SM.4, Question 32.

Age is the participant's age. See Section SM.4, Question 31.

Gender: See Section SM.4, Question 30.

IGI is the score for the Individual-Level Globalization Index. See Section A.1 and SM.5.

Table A.3: Summary of parameters in experimental decisions

Decision	Type of game	Type of interaction	Accounts available to the subject	Parameters of the interaction
1 (Local)	MSC at the Local level	Non-Nested	Personal	
			Local	N=4, MPCR=0.5 MSR=2
2 (National)	MSC at the Local/National level	Nested	Personal	
			Local	N=4, MPCR=0.5 MSR=2
			National	N=12, MPCR=0.25 MSR=3
3 (Global)	MSC at the Local/Global level	Nested	Personal	
			Local	N=4, MPCR=0.5 MLSR=2
			World	N=12, MPCR=0.25 MSR=3

Note: N=Number of subjects per group; MPCR = Marginal per Capita Returns; MSR= Marginal Social Returns

Table A.4: Cronbach's alpha of social identity measures

	Iran	South Africa	Argentina	Russia	Italy	US	Aggregate
Local	0.85	0.72	0.80	0.64	0.88	0.90	0.78
National	0.83	0.72	0.73	0.56	0.85	0.87	0.71
Global	0.83	0.78	0.79	0.64	0.82	0.81	0.74

Table A.5: Correlation between cooperation and social identity measures

Iran

	Local1 Cooperation	Nation2 Cooperation	World3 Cooperation	Local Social Identity	National Social Identity	Global Social Identity
Local1 Cooperation	1					
Nation2 Cooperation	0.6852	1				
World3 Cooperation	0.4842	0.7014	1			
Local Social Identity	0.0664	0.0925	0.0921	1		
National Social Identity	0.0359	0.1359	0.1175	0.6434	1	
Global Social Identity	0.1249	0.2019	0.3688	0.2828	0.4270	1

South Africa

	Local1 Cooperation	Nation2 Cooperation	World3 Cooperation	Local Social Identity	National Social Identity	Global Social Identity
Local1 Cooperation	1					
Nation2 Cooperation	0.4604	1				
World3 Cooperation	0.3385	0.6988	1			
Local Social Identity	0.1541	0.0144	0.0285	1		

National Social Identity	0.0224	0.0497	0.0511	0.5000	1	
Global Social Identity	-0.0616	0.0219	0.0675	0.3102	0.5774	1

Argentina						
	Local1 Cooperation	Nation2 Cooperation	World3 Cooperation	Local Social Identity	National Social Identity	Global Social Identity
Local1 Cooperation	1,00					
Nation2 Cooperation	0.4904	1				
World3 Cooperation	0.4585	0.5854	1			
Local Social Identity	0.1269	0.0002	-0.0509	1		
National Social Identity	0.0409	0.1286	0.0244	0.4066	1	
Global Social Identity	0.1897	0.2446	0.2959	0.3095	0.4309	1

Russia						
	Local1 Cooperation	Nation2 Cooperation	World3 Cooperation	Local Social Identity	National Social Identity	Global Social Identity
Local1 Cooperation	1					
Nation2 Cooperation	0.3528	1				
World3 Cooperation	0.3806	0.6962	1			
Local Social Identity	0.0174	0.0696	0.0223	1		
National Social Identity	0.0487	0.0224	-0.0135	0.6820	1	
Global Social Identity	0.0604	0.1060	-0.0086	0.4688	0.6132	1

(e) Italy						
	Local1 Cooperation	Nation2 Cooperation	World3 Cooperation	Local Social Identity	National Social Identity	Global Social Identity
Local1 Cooperation	1					
Nation2 Cooperation	0.5565	1				
World3 Cooperation	0.5254	0.5828	1			
Local Social Identity	-0.0259	0.0033	0.0776	1		
National Social Identity	0.0681	0.0212	0.1558	0.4070	1	
Global Social Identity	0.1500	0.2074	0.2658	0.1273	0.1399	1

US						
	Local1 Cooperation	Nation2 Cooperation	World3 Cooperation	Local Social Identity	National Social Identity	Global Social Identity
Local1 Cooperation	1					
Nation2 Cooperation	0.4982	1				
World3 Cooperation	0.4391	0.7395	1			
Local Social Identity	-0.1851	-0.1800	-0.1772	1		
National Social Identity	-0.2860	-0.0940	-0.1876	0.4972	1	
Global Social Identity	-0.0159	0.0669	0.0519	0.2647	0.2875	1

(g) Aggregate

	Local1 Cooperation	Nation2 Cooperation	World3 Cooperation	Local Social Identity	National Social Identity	Global Social Identity
Local1 Cooperation	1					
Nation2 Cooperation	0.5459	1				
World3 Cooperation	0.4777	0.6835	1			
Local Social Identity	0.0141	-0.0160	-0.0270	1		
National Social Identity	-0.0235	0.0213	-0.0132	0.5327	1	
Global Social Identity	0.1133	0.1850	0.1997	0.2937	0.4108	1

Table A.6: Local level analysis-All countries

DEPENDENT VARIABLE	Local Contribution	Local Expectation	Local Contribution	Local Contribution
	(1)	(2)	(3)	(4)
Local Social Identity	0.44	-0.14	0.37	0.46
	(0.50)	(0.40)	(0.51)	(0.51)
National Social Identity	-1.71**	0.01	-1.81***	-1.64**
	(0.56)	(0.43)	(0.45)	(0.57)
Global Social Identity	1.71***	1.32**	1.01*	1.57**
	(0.51)	(0.42)	(0.41)	(0.55)
Local Expectation			0.76***	
			(0.06)	
Individual Globalization Index				2.19*
				(1.08)
Global Awareness Index				-0.32
				(0.48)
City	-0.37	-0.23	-0.34	-0.48
	(0.34)	(0.23)	(0.29)	(0.34)
Gender	-0.39	0.02	-0.42+	-0.34
	(0.25)	(0.19)	(0.22)	(0.25)
Education_medium	-0.16	0.47	-0.46	-0.24
	(0.34)	(0.29)	(0.30)	(0.35)
Education_high	0.21	0.36**	-0.02	0.15
	(0.15)	(0.13)	(0.14)	(0.15)
Age_medium	0.02	-0.19	0.12	0.09
	(0.34)	(0.23)	(0.27)	(0.34)
Age_high_std	-0.08	-0.27*	0.08	-0.02
	(0.15)	(0.12)	(0.13)	(0.15)
Income_medium	-0.33	-0.16	-0.31	-0.42
	(0.30)	(0.27)	(0.27)	(0.30)
Income_high	-0.04	0.06	-0.08	-0.17
	(0.16)	(0.14)	(0.14)	(0.16)
Self Employed	-0.09	-0.20	-0.01	-0.15
	(0.36)	(0.28)	(0.31)	(0.36)
Unemployed	-1.35***	-0.08	-1.35***	-1.38***
	(0.40)	(0.49)	(0.40)	(0.41)
Divorced	0.77*	0.36	0.63*	0.71*
	(0.36)	(0.34)	(0.30)	(0.36)
ZAF_dum	1.41**	0.59	1.19*	1.26*
	(0.53)	(0.59)	(0.53)	(0.56)
ARG_dum	2.10***	2.46***	0.52	2.02***
	(0.56)	(0.46)	(0.51)	(0.57)
RUS_dum	1.83**	1.66***	0.79	1.71**
	(0.61)	(0.46)	(0.50)	(0.61)
ITA_dum	1.45**	1.29**	0.70	1.27*
	(0.53)	(0.48)	(0.46)	(0.54)
USA_dum	3.10***	1.99***	1.77***	2.93***
	(0.51)	(0.43)	(0.46)	(0.52)
Constant	5.56***	3.75***	2.75***	5.15***
	(0.58)	(0.49)	(0.56)	(0.70)
Observations	986	971	964	978
Unconstrained obs.	714	829	697	706

Right-censored obs.	219	113	215	219
Left-censored obs.	53	29	52	53
chi2	97.10	94.58	305.0	103.4

Note: Tobit model truncated at the lowest and highest possible levels of contribution (or expectation in column 2). See Appendix: Section A.3 for description of the Tobit estimator. Heteroschedasticity-robust standard errors clustered at the session level are in parenthesis.

*** p<0.001, ** p<0.01, * p<0.05, + p<0.10.

Table A.7: National level analysis-All countries

DEPENDENT VARIABLE	National Contribution (1)	National Expectation (2)	National Contribution (3)	National Contribution (4)
Local Social Identity	-0.66	-0.61	-0.47	-0.71
	(0.45)	(0.43)	(0.40)	(0.45)
National Social Identity	0.25	1.07*	-0.12	0.28
	(0.53)	(0.50)	(0.47)	(0.53)
Global Social Identity	1.47***	1.02*	1.10**	1.22**
	(0.36)	(0.41)	(0.34)	(0.38)
National Expectation			0.39***	
			(0.05)	
Individual Globalization Index				1.15
				(0.84)
Global Awareness Index				0.86+
				(0.50)
local1	0.60***	0.36***	0.50***	0.60***
	(0.05)	(0.04)	(0.05)	(0.05)
City	-0.29	-0.14	-0.37*	-0.31
	(0.20)	(0.21)	(0.19)	(0.20)
Gender	0.04	0.03	0.04	0.04
	(0.15)	(0.17)	(0.15)	(0.16)
Education_medium	0.17	0.24	0.10	0.11
	(0.23)	(0.27)	(0.19)	(0.23)
Education_high	0.07	0.12	0.02	0.04
	(0.12)	(0.11)	(0.11)	(0.12)
Age_medium	-0.37+	-0.12	-0.32	-0.34
	(0.21)	(0.20)	(0.20)	(0.21)
Age_high_std	-0.04	-0.24*	0.02	-0.00
	(0.10)	(0.12)	(0.10)	(0.10)
Income_medium	0.19	0.14	0.11	0.17
	(0.21)	(0.29)	(0.19)	(0.22)
Income_high	0.24+	0.29*	0.11	0.20
	(0.13)	(0.14)	(0.12)	(0.14)
Self Employed	0.39	-0.26	0.50*	0.34
	(0.27)	(0.34)	(0.25)	(0.28)
Unemployed	1.01+	0.56	0.84	1.10*
	(0.55)	(0.46)	(0.55)	(0.56)
Divorced	0.30	0.27	0.16	0.26
	(0.29)	(0.31)	(0.27)	(0.28)
ZAF_dum	0.29	0.10	0.41	0.16
	(0.35)	(0.57)	(0.36)	(0.35)
ARG_dum	0.13	1.58***	-0.26	0.05
	(0.37)	(0.42)	(0.36)	(0.36)
RUS_dum	0.42	1.93***	-0.23	0.40
	(0.33)	(0.41)	(0.32)	(0.33)
ITA_dum	0.63+	1.30**	0.22	0.57+
	(0.33)	(0.41)	(0.31)	(0.34)
USA_dum	1.32***	1.92***	0.66+	1.19***
	(0.36)	(0.39)	(0.36)	(0.36)
Constant	-0.18	0.47	-0.62	-0.96
	(0.53)	(0.54)	(0.46)	(0.65)
Observations	985	960	959	977
Unconstrained obs.	803	866	780	795
Right-censored obs.	105	58	104	105

Left-censored obs.	77	36	75	77
chi2	327.5	228.0	516.2	344.9

Note: See Table A.6.

Table A.8: World level analysis-All countries

DEPENDENT VARIABLE	Global Contribution (1)	Global Expectation (2)	Global Contribution (3)	Global Contribution (4)
Local Social Identity	-0.58	-0.51	-0.28	-0.61
	(0.50)	(0.49)	(0.42)	(0.50)
National Social Identity	-0.43	-0.22	-0.26	-0.40
	(0.67)	(0.52)	(0.57)	(0.66)
Global Social Identity	2.13***	1.70***	1.24**	1.94***
	(0.48)	(0.48)	(0.39)	(0.48)
World Expectation			0.50***	
			(0.04)	
Individual Globalization				0.92
				(0.97)
Global Awareness Index				0.67
				(0.52)
local1	0.55***	0.31***	0.40***	0.55***
	(0.05)	(0.05)	(0.04)	(0.05)
City	-0.64**	0.01	-0.68**	-0.67**
	(0.23)	(0.25)	(0.22)	(0.23)
Gender	-0.16	-0.23	-0.10	-0.16
	(0.18)	(0.19)	(0.16)	(0.18)
Education_medium	0.27	0.67*	0.05	0.21
	(0.21)	(0.29)	(0.20)	(0.22)
Education_high	0.25*	0.17	0.19	0.23+
	(0.13)	(0.13)	(0.12)	(0.13)
Age_medium	-0.33	-0.32	-0.22	-0.31
	(0.21)	(0.24)	(0.18)	(0.22)
Age_high_std	-0.20+	-0.26*	-0.13	-0.18
	(0.11)	(0.13)	(0.10)	(0.11)
Income_medium	0.42	0.15	0.39	0.41
	(0.26)	(0.32)	(0.25)	(0.27)
Income_high	0.23+	0.22	0.13	0.19
	(0.14)	(0.15)	(0.13)	(0.16)
Self Employed	0.19	0.12	0.13	0.16
	(0.35)	(0.35)	(0.30)	(0.35)
Unemployed	0.88	-0.14	0.97*	0.96+
	(0.55)	(0.53)	(0.47)	(0.58)
Divorced	0.53	0.70*	0.27	0.50
	(0.32)	(0.32)	(0.30)	(0.32)
ZAF_dum	0.17	0.33	0.14	0.06
	(0.39)	(0.61)	(0.42)	(0.40)
ARG_dum	-0.31	1.48***	-0.96*	-0.37
	(0.39)	(0.43)	(0.41)	(0.39)
RUS_dum	0.35	2.28***	-0.73+	0.34
	(0.38)	(0.46)	(0.40)	(0.38)
ITA_dum	0.23	1.36**	-0.42	0.16
	(0.35)	(0.46)	(0.35)	(0.36)
USA_dum	1.06*	1.90***	0.25	0.96*
	(0.45)	(0.43)	(0.48)	(0.46)
Constant	0.55	1.25*	-0.29	-0.08
	(0.57)	(0.53)	(0.52)	(0.70)

Observations	984	954	952	976
Unconstrained obs.	772	841	744	764
Right-censored obs.	109	74	105	109
Left-censored obs.	103	39	103	103
chi2	241.6	212.4	489.1	245.1

Note: See note to Table A.6.

Table A.9: Local level analysis-Individual countries

DEP VAR: Contributions to Local account (Dec. 1)	IRN (1)	ZAF (2)	ARG (3)	RUS (4)	ITA (5)	USA (6)
Local Social Identity	1.59	2.66**	1.25	0.52	-0.98	-0.91
	(2.05)	(0.88)	(1.09)	(1.20)	(1.31)	(1.40)
National Social Identity	-0.39	-0.78	-1.84	-0.92	0.31	-3.69*
	(2.29)	(1.15)	(1.37)	(1.63)	(1.44)	(1.55)
Global Social Identity	2.64	0.14	2.37*	1.75+	3.04*	0.45
	(1.75)	(1.00)	(1.05)	(1.03)	(1.35)	(1.38)
Gender	0.56	-1.14*	0.22	-1.08*	-0.28	-0.68
	(0.73)	(0.47)	(0.49)	(0.55)	(0.56)	(0.63)
Education_medium	-0.03	0.94	-0.62	-0.47	-0.25	-0.05
	(1.86)	(0.60)	(0.62)	(0.67)	(0.80)	(0.89)
Education_high	0.78	0.25	-0.29	0.47	-0.01	0.14
	(0.50)	(0.27)	(0.33)	(0.34)	(0.47)	(0.36)
Age_medium	-1.12	0.28	1.32*	0.82	1.40*	-1.04
	(1.00)	(0.48)	(0.64)	(0.69)	(0.67)	(0.78)
Age_high_std	0.17	0.19	0.42	0.10	0.15	-0.25
	(0.54)	(0.28)	(0.31)	(0.30)	(0.34)	(0.39)
Income_medium	-1.14	-0.38	1.00	0.21	0.70	-0.52
	(0.78)	(0.49)	(0.84)	(0.68)	(0.67)	(0.70)
Income_high	-1.44	0.26	0.31	0.31	0.27	-0.29
	(0.99)	(0.22)	(0.38)	(0.35)	(0.39)	(0.50)
Constant	2.51+	5.17***	4.89***	6.42***	4.41**	12.03***
	(1.52)	(0.93)	(1.29)	(0.98)	(1.48)	(1.21)
Observations	156	121	190	193	185	163
Unconstrained obs.	106	108	143	142	134	96
Right-censored obs.	24	11	38	47	42	63
Left-censored obs.	26	2	9	4	9	4
chi2	18.97	28.83	15.51	16.27	15.59	23.13

Note: Replication of the model as of Table A.6, column 1, for individual countries. See note to Table A.6. Due to lower sample size at the country level, robust standard errors were not clustered at the session level, but were robust to heteroschedastity according to the Eicker-Huber-White method (White, 1980).

Table A.10: National level analysis-Individual countries

DEP VAR: Contributions to National account (Dec. 2)	IRN<br>(1)	ZAF<br>(2)	ARG<br>(3)	RUS<br>(4)	ITA<br>(5)	USA<br>(6)
Local Social Identity	-0.10	-0.20	-1.39	1.42	0.09	-2.35+
	(1.23)	(1.00)	(0.92)	(1.06)	(0.97)	(1.30)
National Social Identity	2.21	-0.55	1.26	-2.33	-0.28	1.40
	(1.53)	(1.01)	(1.14)	(1.45)	(0.84)	(1.29)
Global Social Identity	1.52+	1.34	1.40+	2.07*	1.64+	1.70
	(0.88)	(0.86)	(0.78)	(0.86)	(0.95)	(1.22)
local1	0.84***	0.38***	0.59***	0.37***	0.58***	0.83***
	(0.11)	(0.10)	(0.10)	(0.10)	(0.08)	(0.13)
Gender	-0.44	-0.51	0.26	-0.64	0.41	0.52
	(0.42)	(0.32)	(0.40)	(0.45)	(0.37)	(0.55)
Education_medium	0.46	0.43	0.70	0.15	-0.24	-0.23
	(0.91)	(0.35)	(0.46)	(0.52)	(0.55)	(0.72)
Education_high	-0.42	0.14	0.17	0.31	-0.08	0.10
	(0.33)	(0.22)	(0.29)	(0.30)	(0.30)	(0.32)
Age_medium	0.10	-0.26	0.09	-0.36	-0.75	-0.07
	(0.65)	(0.45)	(0.55)	(0.56)	(0.46)	(0.69)
Age_high_std	-0.29	-0.04	0.20	-0.14	-0.06	-0.05
	(0.37)	(0.17)	(0.28)	(0.27)	(0.22)	(0.33)
Income_medium	0.20	0.40	0.49	-0.01	0.31	-0.03
	(0.51)	(0.33)	(0.79)	(0.55)	(0.45)	(0.63)
Income_high	-0.35	0.25	0.32	0.06	0.42	-0.14
	(0.66)	(0.22)	(0.35)	(0.28)	(0.28)	(0.36)
Constant	-3.31***	1.80*	-0.80	2.17*	0.40	-0.58
	(0.97)	(0.75)	(1.16)	(0.91)	(0.95)	(1.59)
Observations	155	121	190	193	185	163
Unconstrained obs.	109	115	158	164	159	117
Right-censored obs.	13	5	15	16	18	40
Left-censored obs.	33	1	17	13	8	6
chi2	102.8	48.07	60.56	31.05	88.77	54.15

Note: Replication of the model as of Table A.7, column 1 for individual countries. See notes to Table A.6 and A.9.

Table A.11: World level analysis-Individual countries

DEP VAR: Contributions to World account (Dec. 3)	IRN<br>(1)	ZAF<br>(2)	ARG<br>(3)	RUS<br>(4)	ITA<br>(5)	USA<br>(6)
Local Social Identity	0.47	-0.07	-2.28*	0.96	0.65	-1.54
	(1.30)	(1.04)	(0.92)	(1.21)	(1.10)	(1.61)
National Social Identity	0.13	-0.55	-1.28	-1.20	1.18	-1.52
	(1.79)	(1.19)	(1.17)	(1.72)	(1.13)	(1.71)
Global Social Identity	5.60***	1.74*	3.49***	-0.28	2.22*	2.50+
	(1.19)	(0.86)	(0.97)	(1.06)	(1.09)	(1.47)
local1	0.51***	0.34**	0.62***	0.49***	0.59***	0.79***
	(0.12)	(0.13)	(0.12)	(0.11)	(0.09)	(0.15)
Gender	-0.18	0.03	-0.88+	0.11	0.12	0.34
	(0.52)	(0.38)	(0.46)	(0.47)	(0.42)	(0.62)
Education_medium	-0.79	-0.09	0.86	0.51	-0.02	-0.34
	(1.27)	(0.43)	(0.62)	(0.55)	(0.55)	(0.90)
Education_high	0.08	0.24	0.60*	0.09	-0.17	0.28
	(0.33)	(0.24)	(0.30)	(0.30)	(0.32)	(0.35)
Age_medium	-0.78	0.02	-0.54	-0.29	-0.53	0.99
	(0.71)	(0.51)	(0.61)	(0.60)	(0.52)	(0.79)
Age_high_std	-0.96**	0.14	-0.33	-0.08	0.22	-0.06
	(0.37)	(0.21)	(0.31)	(0.27)	(0.24)	(0.40)
Income_medium	0.81	-0.30	0.21	0.38	0.65	0.35
	(0.61)	(0.34)	(0.82)	(0.57)	(0.45)	(0.73)
Income_high	0.25	-0.05	-0.19	0.09	0.29	0.36
	(0.61)	(0.22)	(0.39)	(0.29)	(0.31)	(0.49)
Constant	-2.08+	1.65+	1.51	1.63	-2.06+	0.29
	(1.10)	(0.86)	(1.28)	(1.00)	(1.15)	(1.77)
Observations	154	121	190	193	185	163
Unconstrained obs.	109	113	144	162	150	110
Right-censored obs.	11	5	17	20	19	41
Left-censored obs.	34	3	29	11	16	12
chi2	70.93	27.18	57.07	25.40	79.85	47.53

Note: Replication of the model as of Table A.8, column 1 for individual countries. See notes to Table A.6 and A.9.

Table A.12: Interaction between GSI and countries

DEP VAR: Contributions to level	LOCAL<br>(1)	NATIONAL<br>(2)	GLOBAL<br>(3)
Local Social Identity	0.47	-0.53	-0.37
	(0.49)	(0.49)	(0.48)
National Social Identity	-1.60**	-0.27	-0.82
	(0.55)	(0.55)	(0.71)
Global Social Identity	3.60**	3.64**	6.78***
	(1.40)	(1.34)	(1.43)
ZAF_dum_X_Global Social Identity	-2.65+	-2.26	-4.73**
	(1.57)	(1.60)	(1.66)
ARG_dum_X_Global Social Identity	-1.52	-1.21	-3.40*
	(1.79)	(1.48)	(1.61)
RUS_dum_X_Global Social Identity	-2.24	-1.99	-6.33***
	(1.72)	(1.51)	(1.55)
ITA_dum_X_Global Social Identity	-0.55	-0.44	-2.79+
	(1.89)	(1.63)	(1.60)
USA_dum_X_Global Social Identity	-4.39**	-2.26	-5.54**
	(1.70)	(1.71)	(1.95)
ZAF_dum	2.58**	1.96*	2.83**
	(0.86)	(0.90)	(0.93)
ARG_dum	2.68**	1.55*	1.93*
	(0.85)	(0.77)	(0.84)
RUS_dum	2.76**	2.06*	3.98***
	(0.95)	(0.91)	(0.91)
ITA_dum	1.30	1.11	1.61+
	(1.02)	(0.95)	(0.90)
USA_dum	5.34***	3.74***	4.87***
	(0.94)	(0.94)	(1.19)
City	-0.36	-0.42	-0.77**
	(0.34)	(0.27)	(0.29)
Gender	-0.37	-0.14	-0.28
	(0.25)	(0.19)	(0.21)
Education_medium	-0.10	0.14	0.25
	(0.34)	(0.27)	(0.24)
Education_high	0.21	0.15	0.31*
	(0.15)	(0.14)	(0.15)
Age_medium	-0.01	-0.34	-0.35
	(0.34)	(0.26)	(0.23)
Age_high_std	-0.08	-0.06	-0.21+
	(0.15)	(0.12)	(0.12)
Income_medium	-0.30	0.09	0.30
	(0.31)	(0.25)	(0.27)
Income_high	-0.05	0.22	0.17
	(0.16)	(0.14)	(0.14)
Self Employed	-0.12	0.35	0.18
	(0.37)	(0.33)	(0.38)
Unemployed	-1.33***	0.45	0.34

	(1)	(2)	(3)
	(0.38)	(0.51)	(0.54)
Divorced	0.77*	0.65+	0.82*
	(0.37)	(0.33)	(0.35)
Constant	4.68***	2.43**	1.65+
	(0.71)	(0.77)	(0.86)
Observations	986	986	986
Unconstrained obs.	714	804	774
Right-censored obs.	219	105	109
Left-censored obs.	53	77	103
chi2	108.3	136.9	181.5

Note: The models of columns 1, 2, and 3 are equivalent to those of Table A.6, column 1, Table A.7, column 2, and Table A.8, column 3, after introducing interaction terms between country and the GSI index. Interaction terms are labelled "Country_Name_X_Global Social Identity", where Country_Name={ZAF (South Africa); ARG (Argentina), RUS (Russia), ITA, (Italy), USA}. See notes to Table A.6 for description of model characteristics.

Table A.13: Results of Wald tests on null hypothesis that GSI coefficients differ in pairs of countries – Local level

	Iran	South Africa	Argentina	Russia	Italy
South Africa	2.65+				
	(1.57)				
Argentina	1.52	-1.13			
	(1.79)	(1.33)			
Russia	2.24	-.41	.72		
	(1.72)	(1.22)	(1.54)		
Italy	.55	-2.1	-.97	-1.69	
	(1.89)	(1.51)	(1.75)	(1.65)	
US	4.39**	1.74	2.87+	2.15	3.84*
	(1.7)	(1.21)	(1.5)	(1.4)	(1.65)

Note: Results of Wald tests over the null hypotheses that coefficients for the interaction term between the GSI index and a pair of countries are equal to each other. The two cells at the intersection of the country indicated in the column and the country indicated in the row of the tables report the difference of the coefficients (top cell) and the robust standard error (in square bracket). The coefficient is the difference between the coefficient for the country in the column and the coefficient for the country in the row of the table. A positive (negative) value denotes a larger effect for GSI in the country indicated in the column (row) of the table. Wald tests have been run on the regression as of Table A.12, column 1. *** p<0.001, ** p<0.01, * p<0.05, + p<0.10.

Table A.14: Results of Wald tests on null hypothesis that GSI coefficients differ in pairs of countries – National level

	Iran	South Africa	Argentina	Russia	Italy
South Africa	2.26				
	(1.6)				
Argentina	1.21	-1.05			
	(1.48)	(1.12)			
Russia	1.99	-0.27	0.78		
	(1.51)	(1.19)	(1.)		
Italy	0.44	-1.82	-0.77	-1.55	
	(1.63)	(1.34)	(1.19)	(1.24)	
US	2.26	0.00	1.05	0.27	1.82
	(1.71)	(1.41)	(1.26)	(1.32)	(1.43)

Note: See notes to Table A.13. Wald tests have been run on the regression as of Table A.12, column 2. *** p<0.001, ** p<0.01, * p<0.05, + p<0.10.

Table A.15: Results of Wald tests on null hypothesis that GSI coefficients differ in pairs of countries – Global level

	Iran	South Africa	Argentina	Russia	Italy
South Africa	4.73**				
	(1.66)				
Argentina	3.40*	-1.33			
	(1.61)	(1.12)			
Russia	6.33***	1.60	2.93**		
	(1.55)	(1.05)	(.98)		
Italy	2.79+	-1.93+	-.61	-3.530***	
	(1.6)	(1.17)	(1.1)	(1.01)	
US	5.54**	0.82	2.15	-.78	2.75+
	(1.95)	(1.53)	(1.54)	(1.44)	(1.57)

Note: See notes to Table A.13. Wald tests have been run on the regression as of Table A.12, column 3. *** p<0.001, ** p<0.01, * p<0.05, + p<0.10.

Table A.16: Pooled regression

DEPENDENT VARIABLE	Contribution (1)	Expectation (2)	Contribution (3)	Contribution (4)	Contribution (5)
Local Social Identity	-0.19	-0.36	-0.01	-0.19	-0.20
	(0.38)	(0.35)	(0.36)	(0.38)	(0.39)
National Social Identity	-1.04*	0.03	-1.12**	-1.04*	-0.98*
	(0.46)	(0.40)	(0.37)	(0.46)	(0.47)
Global Social Identity	2.26***	1.62***	1.38***	2.71***	2.01***
	(0.37)	(0.37)	(0.29)	(0.44)	(0.41)
Expectation			0.62***		
			(0.04)		
Global Social Identity_X_Local				-1.18*	
				(0.47)	
Global Social				-0.18	
				(0.34)	
Individual Globalization Index					2.05*
					(0.90)
Global Awareness Index					0.36
					(0.43)
City	-0.51+	-0.17	-0.51*	-0.51+	-0.60*
	(0.27)	(0.22)	(0.22)	(0.27)	(0.27)
Gender	-0.29	-0.13	-0.25+	-0.29	-0.26
	(0.18)	(0.17)	(0.15)	(0.18)	(0.18)
Education_medium	0.05	0.43+	-0.14	0.05	-0.04
	(0.24)	(0.26)	(0.20)	(0.24)	(0.24)
Education_high	0.24+	0.26*	0.11	0.24+	0.18
	(0.13)	(0.12)	(0.11)	(0.13)	(0.13)
Age_medium	-0.20	-0.21	-0.11	-0.21	-0.14
	(0.23)	(0.21)	(0.18)	(0.23)	(0.23)
Age_high_std	-0.12	-0.26*	0.00	-0.12	-0.06
	(0.11)	(0.11)	(0.10)	(0.11)	(0.11)
Income_medium	0.03	-0.03	-0.01	0.03	-0.04
	(0.21)	(0.25)	(0.19)	(0.21)	(0.21)
Income_high	0.14	0.18	0.03	0.14	0.04
	(0.12)	(0.12)	(0.11)	(0.12)	(0.13)
Self Employed	0.14	-0.14	0.18	0.14	0.08
	(0.30)	(0.27)	(0.25)	(0.30)	(0.30)
Unemployed	-0.17	-0.10	-0.11	-0.17	-0.12
	(0.33)	(0.42)	(0.31)	(0.33)	(0.34)
Divorced	0.75*	0.58*	0.47+	0.75*	0.68*
	(0.29)	(0.27)	(0.25)	(0.29)	(0.28)
South Africa	1.03**	0.63	0.88*	1.03**	0.85*
	(0.39)	(0.56)	(0.38)	(0.39)	(0.40)
Argentina	1.24***	2.23***	0.10	1.24***	1.14**
	(0.37)	(0.41)	(0.31)	(0.37)	(0.38)
Russia	1.35**	2.29***	0.10	1.35**	1.27**
	(0.45)	(0.42)	(0.36)	(0.45)	(0.46)
Italy	1.14**	1.58***	0.33	1.15**	0.99*
	(0.41)	(0.43)	(0.34)	(0.41)	(0.42)
USA	2.69***	2.47***	1.36***	2.69***	2.50***
	(0.45)	(0.39)	(0.41)	(0.45)	(0.45)
National level	-2.06***	-0.80***	-1.63***	-2.60***	-2.06***
	(0.11)	(0.09)	(0.11)	(0.27)	(0.11)

World level	-2.19***	-0.68***	-1.87***	-2.82***	-2.20***
	(0.13)	(0.13)	(0.13)	(0.29)	(0.13)
Constant	5.46***	3.51***	3.30***	5.85***	4.70***
	(0.51)	(0.44)	(0.45)	(0.53)	(0.65)
Observations	2,958	2,899	2,878	2,958	2,934
chi2	606.2	309.3	657.3	702.8	629.9

Note: Tobit panel regression with the individual as the cross-section component and the level of decision (local, national, or global) as the "panel" component. Heteroschedasticity-robust standard errors are clustered at the individual level.

Table A.17: Analysis of optimism

DEPENDENT VARIABLE	(1)<br>Optimism_Local1	(2)<br>Optimism_Nation2	(3)<br>Optimism_World3	(4)<br>Optimism_Mean
Local Social Identity	0.07	-0.51	-0.42	-0.31
	(0.36)	(0.37)	(0.42)	(0.32)
National Social Identity	0.01	0.67	-0.49	0.06
	(0.37)	(0.46)	(0.47)	(0.35)
Global Social Identity	1.03**	1.30***	1.83***	1.31***
	(0.36)	(0.37)	(0.41)	(0.32)
City	-0.04	-0.15	-0.02	-0.09
	(0.22)	(0.22)	(0.24)	(0.19)
Gender	0.02	-0.08	-0.29	-0.13
	(0.17)	(0.17)	(0.18)	(0.15)
Education_medium	0.60*	0.15	0.58*	0.47*
	(0.25)	(0.26)	(0.27)	(0.23)
Education_high	0.29*	0.16	0.19	0.21*
	(0.12)	(0.12)	(0.12)	(0.10)
Age_medium	-0.15	-0.08	-0.28	-0.19
	(0.20)	(0.20)	(0.23)	(0.18)
Age_high_std	-0.23*	-0.22+	-0.23+	-0.25*
	(0.10)	(0.11)	(0.12)	(0.10)
Income_medium	-0.18	0.08	0.08	0.03
	(0.22)	(0.26)	(0.29)	(0.22)
Income_high	-0.06	0.27*	0.21	0.18+
	(0.12)	(0.13)	(0.14)	(0.10)
Self Employed	-0.11	-0.30	0.05	-0.16
	(0.25)	(0.31)	(0.31)	(0.24)
Unemployed	-0.11	0.26	-0.39	-0.07
	(0.44)	(0.43)	(0.48)	(0.37)
Divorced	0.08	0.50+	0.80**	0.52*
	(0.28)	(0.29)	(0.29)	(0.24)
South Africa	-0.48	-0.38	0.50	-0.17
	(0.52)	(0.57)	(0.59)	(0.50)
Argentina	0.76+	0.96*	1.74***	1.11**
	(0.40)	(0.41)	(0.40)	(0.35)
Russia	-0.10	0.93*	2.43***	1.02**
	(0.40)	(0.41)	(0.46)	(0.37)
Italy	-0.14	0.15	1.54***	0.52
	(0.44)	(0.40)	(0.44)	(0.39)
USA	-0.80*	-0.09	2.30***	0.47
	(0.37)	(0.37)	(0.39)	(0.34)
Constant	-1.19**	-0.68	-1.24**	-0.99**
	(0.42)	(0.42)	(0.43)	(0.36)
Observations	971	967	961	985
R-squared	0.07	0.08	0.16	0.10

Note: OLS regression with heteroschedasticity-robust standard errors clustered at the session level.

Table A.18: Analysis of Forecast error

VARIABLES	(1)<br>FE_Local1	(2)<br>FE_Nation2	(3)<br>FE_World3	(4)<br>FE_Mean
Local Social Identity	-0.21	0.48*	0.01	0.10
	(0.23)	(0.21)	(0.20)	(0.16)
National Social Identity	-0.15	-0.28	-0.46+	-0.27
	(0.25)	(0.25)	(0.24)	(0.18)
Global Social Identity	0.07	0.29	0.80***	0.40*
	(0.19)	(0.25)	(0.21)	(0.17)
City	-0.11	-0.09	-0.05	-0.10
	(0.13)	(0.12)	(0.13)	(0.09)
Gender	0.10	0.12	0.12	0.11
	(0.10)	(0.09)	(0.10)	(0.07)
Education_medium	-0.21+	-0.23+	-0.13	-0.20*
	(0.12)	(0.13)	(0.15)	(0.09)
Education_high	-0.15*	-0.13*	-0.18**	-0.15**
	(0.06)	(0.06)	(0.07)	(0.05)
Age_medium	0.09	0.21	0.17	0.15
	(0.11)	(0.13)	(0.14)	(0.10)
Age_high_std	0.03	0.02	0.03	0.04
	(0.05)	(0.06)	(0.07)	(0.04)
Income_medium	0.18	0.03	-0.21	0.03
	(0.12)	(0.13)	(0.15)	(0.10)
Income_high	0.03	-0.02	-0.07	-0.01
	(0.07)	(0.07)	(0.08)	(0.06)
Self Employed	0.03	0.16	0.41*	0.19
	(0.14)	(0.17)	(0.18)	(0.13)
Unemployed	0.16	0.05	0.15	0.14
	(0.22)	(0.21)	(0.25)	(0.17)
Divorced	0.27+	0.12	0.18	0.18
	(0.16)	(0.15)	(0.18)	(0.13)
South Africa	-0.55+	-0.47+	-0.80**	-0.63**
	(0.29)	(0.24)	(0.25)	(0.20)
Argentina	-0.97***	-0.46*	-0.93***	-0.78***
	(0.23)	(0.21)	(0.24)	(0.18)
Russia	-0.59*	-0.19	-0.50*	-0.44*
	(0.26)	(0.23)	(0.23)	(0.19)
Italy	-0.60*	-0.67**	-0.92***	-0.75***
	(0.25)	(0.23)	(0.23)	(0.18)
USA	-0.47*	-0.54*	-0.86***	-0.66***
	(0.22)	(0.22)	(0.23)	(0.18)
Constant	2.78***	2.30***	3.13***	2.72***
	(0.26)	(0.28)	(0.27)	(0.21)
Observations	971	967	961	985
R-squared	0.05	0.04	0.06	0.07

Note: See note to Table A.13.

Supplementary Figures

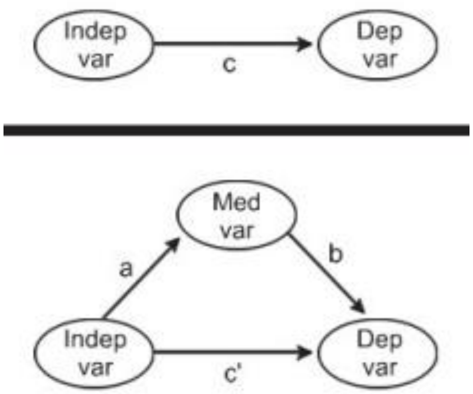

Figure A.1: Description of mediation effect

Note: c is the Total Effect exerted by the Independent variable onto the DV. When a Mediating variable is introduced in the model, the Indirect Effect is given by the path ab, where a is the effect of the Independent variable onto the Mediating variable and b is the effect of the Mediating variable onto the DV. c' is the Direct effect, i.e., the effect of the Independent variable exerted directly onto the DV while the Mediating variable is included in the model.

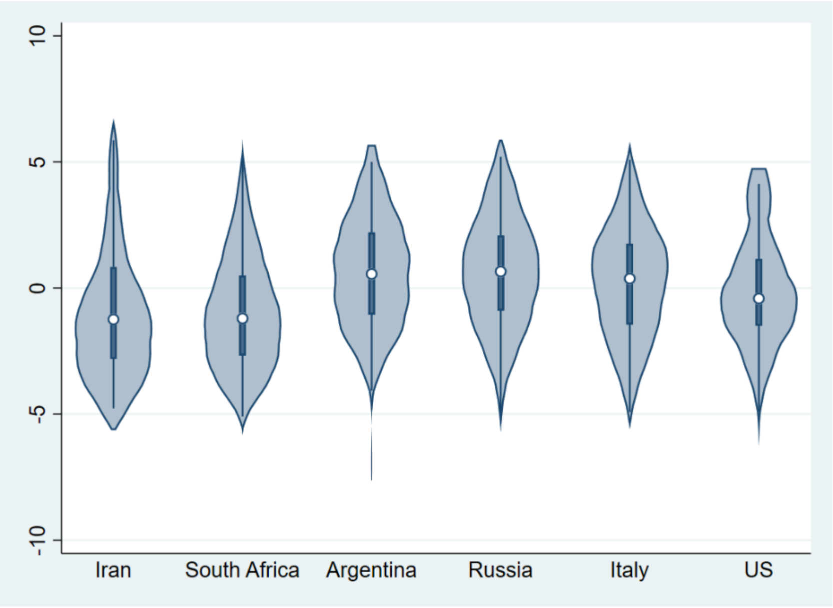

Figure A.2: Distribution of Optimism per country (mean over Local 1, National 2, World 3)

Note: Distribution of the mean level of optimism regarding contributions to the Local (Decision 1), National (Decision 2), and World (Decision 3) accounts. Plots include a white point indicating the median of the distribution, a box for the interquartile range (from 25th percentile up to 75th), and spikes extending to the upper- and lower-adjacent values. Overlaid with this box plot is the estimated k-density.

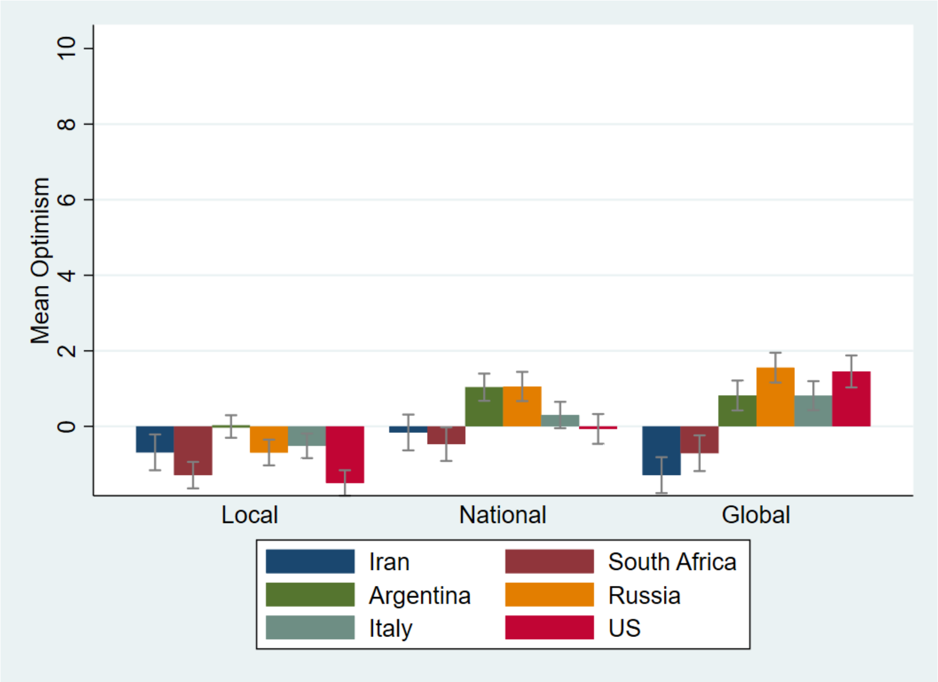

Figure A.3: Mean optimism by country and decision
Note: Capped bars plot 95% confidence intervals for the mean.

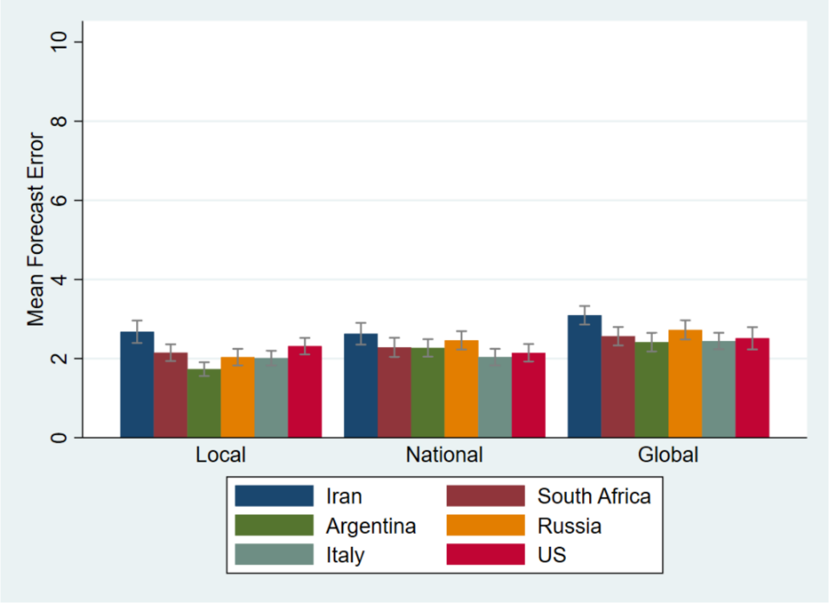

Figure A.4: Mean forecast error by country and decision
Note: Capped bars plot 95% confidence intervals for the mean.

Supplementary Methods

SM.1: Choice of Locations and Recruitment Procedures

Within each country the research focused on a large metropolitan area and on surrounding areas that were likely to be less globalized in nature. For example, in Russia the research occurred in Kazan, a globalized city in Tatarstan, and in more rural surrounding outposts (see pictures below). In the United States the metropolitan area was Columbus, Ohio; in South Africa it was Johannesburg; in Italy it was Milan; in Argentina it was Buenos Aires; and in Iran it was Tehran.

Research participants were drawn from the general population. A quota sampling recruitment method was used based on three characteristics: gender; age (18- 30, 31-50, 51-70); socioeconomic status (low, medium, high). These characteristics produced an 18 cell matrix; a target quota of ten or eleven citizens per cell was to be recruited within each country, yielding approximately 190 participants per country. All participants possessed at least a 4th grade reading level, had lived in the given locality for at least one year, and were citizens of the country studied. Descriptive statistics of the samples are reported in Table A.2.

Recruitment methods varied locally according to what was considered most appropriate by a local collaborator. In three countries (Argentina, Italy and Russia) recruitment was subcontracted to survey agencies specialized in market research. In the other countries people were recruited through other methods such as distribution of posters, leaflets, or advertisements on newspapers, and personal phone solicitation.

SM.2: Summary of Experiment Protocol

The research was conducted between May and September 2006. Local researchers were native to the place where the research was conducted. Experiment control was guaranteed by the presence of a member of the core research team during all experimental sessions in each location.

An experiment session lasted around an hour, and comprised three experimental decisions and the completion of the questionnaire. Participants were paid the purchasing power equivalent of US$8.00 as a show-up fee as they entered the experiment room. Participants randomly chose an identification number to identify themselves throughout the experiment; never were participants' names or other personally identifying information provided to the researchers. Furthermore, to guard against any possible political risk to participants in Iran and Russia, recruitment lists were destroyed at the beginning of each experimental session in view of participants.

To the extent possible, subjects were isolated from one another so that privacy was maintained throughout the experiment. Instructions for the tasks were delivered orally by a native speaker of the language in which the experiment was conducted. A written comprehension check consisting of three questions regarding the basic logic and procedure of the Multi-level Sequential Cooperation (hereafter MSC) game was administered to subjects after providing instructions for the decision. This was collected and saved by the experimenter so that decisions of subjects who had failed the test could be expunged from the dataset at the end of the session. Shorter comprehension checks were also conducted after the instructions for second and third decisions to make certain

subjects understood the basic logic of the MSC game. The correct answers to the questions were communicated before subjects made their choices.

Subjects made three experimental decisions in a fixed order. Pilot tests found no ordering effects. Decisions were anonymous, and the groups to which subjects were assigned were randomly selected at the beginning of each decision. No feedback between decisions was provided. Hence, the three decisions can be treated as independent. The first decision (Decision L) measured propensity to cooperate with people living in the same locality through a non-nested MSC. The next two decisions (Decision N and Decision W) examined how much individuals were willing to cooperate beyond their locality with people coming from other areas of their nation and other parts of the world. We used an MSC experiment at the national and global level for this purpose with identical monetary incentives. The experimental script is reported in section SM.3. The type of interaction and the parameters for each decision are summarized in Table A.3.

Decisions were made privately using tokens that could be allocated into envelopes representing the personal, local, national and global accounts. In Iran, due to some logistical impediments, subjects made their choices with pen and paper. After subjects completed the three decisions there was a waiting period while their outcomes were determined. It was during this waiting period that subjects completed the questionnaire from which the social identity measures and additional demographic information were derived. When necessary, the questionnaire was read aloud as participants followed along and made their responses. Average take-home earnings from the experiment were the purchasing power equivalent of US$34.00.

Section SM.4 reports the questions relevant for the analyses reported in this paper. The whole version of the questionnaire can be found in Buchan et al. (2009: SOM). Other questions (not reported) measured an individual’s attitudes towards global processes, such as the existence of ethnocentric attitudes, specifically, the participant’s willingness to restrict migrants’ access, and the necessity to protect national culture from foreign influence. Additional questions inquired about a participant’s opinions on international trade and migration.

SM.3: Experiment Script (Version used in a US location)

<Note: Instructions to experimenters are in italics. Text in non-italics is read to the Ss.>

As Ss arrive, welcome them and hand them a consent form. If they cannot read the Consent form, then have it read to them. Typically this form will be read outside the experiment room. The Consent form notes that if they enter the room, they are agreeing to participate; Ss will not sign the form, only non-verbal consent is necessary. Local custom will dictate what should be done.

If Ss refuse to participate, then pay the show-up fee and send them on their way.

Once Ss are seated, pay them their show-up fee. Once paying each, have the Ss randomly draw a sheet of stickers with their ID #. (The stickers may be placed down on a table to allow Ss to randomly draw them).

First please take your show up fee out of the envelope and put it away, this money is yours to keep for coming to the experiment. Next, please draw a sheet of stickers. These stickers will have your ID number on them. You will notice you have 12 stickers on the sheet. These will be used later for your decisions in the experiment; you will put the ID stickers on different pieces of paper and on envelopes.

Now please place one ID sticker on the empty envelope. We will collect these envelopes now and at the end of the experiment, each envelope will be returned – containing the experiment earnings - to the person with the matching ID number. We will not know who you are or what decisions you made – we will only know your ID number.

The experimenter should collect the envelopes.

Please turn your sheet over and do not show it to the others. Do not tell anyone, except the experimenter, your number. Please wait until everyone has arrived and then we can get started. Please keep quiet and do not speak to the others in this room.

When everyone has arrived and is seated then the instructions can begin.

Welcome to this research project. An international team of researchers is looking at the way in which people in this <local community>, this <COUNTRY NAME> and around the world make decisions. If you pay close attention to the instructions then you could make a significant amount of money.

The research team that is here today includes myself <give name>, an assistant <give name> and <give name of Core member> along with another assistant <give name> who will be outside the room making your payments.

Everyone should be present in the room at this point and acknowledge the introduction. The Core member and the second assistant should then leave.

In this project you are going to be asked to make decisions with other people. Some will be in this local community, but they may not be in this room now; some will be from this <COUNTRY NAME> and some will be from countries around the world. Many people have already made their decisions and other groups are doing the same research this week. Your choices, and the choices by others, will be matched with the help of a colleague at another university when you are finished. You will be paid in cash at the end of this research for the decisions that you and the people you have been matched with made.

The same instructions are being given to other people in other countries. That is why we are reading this script. Everyone is hearing the same thing you are, except in their own language.

All of the decisions are similar, so please pay attention to these instructions. At the outset of each decision you will be given 10 colored tokens. Everyone will get the same materials that you get. It will be important to keep in mind that colored tokens are worth <$.50> each to you. For other people, whether from around here, in the region or around the world, their colored tokens also are worth money to them. We have taken care that their tokens, once converted to their foreign currency, are worth the same value as your tokens in terms of what could be purchased with them. That is, people in other countries will receive an amount in their currency such that they can buy in their country the same amount of goods that <*$.50*> will buy in the United States.

Again, keep in mind that you are being matched with other people (some of whom are from around here and some of whom are from around the world). What those people have decided to do and what you will decide to do affects how much you can make. When your decisions are submitted, our core team member will be using our computer connection to receive information about others' choices in order to calculate each person's payments. This may take a little while so please be prepared to wait for a few minutes at the end of the session so that we can give you your final payment before you leave today.

After a pause, begin the instructions…

Your task is to decide how you want to allocate your tokens between different envelopes. You will have several options, sometimes two and sometimes three. Here I will explain the simplest decision where there are only two ways to allocate your tokens.

DECISION ONE.

In first decision you will be given 10 tokens, and you can put your tokens into your "Personal" envelope or into your <Local> envelope. The number of tokens you put into any envelope is entirely up to you.

What's the difference between the envelopes? Whatever you put into the "Personal" envelope is yours and will not be shared with anyone else. As mentioned before, for every colored token you put into that envelope is worth <$.50> to you regardless of the other people's decisions. Now, what about the <Local> envelope? Any colored tokens that you and three other people put into your <Local> envelopes will be doubled by me. You and the other three people will get an equal share of that amount.

Where do these three other people come from? As I mentioned, you are going to make this decision with 3 other people. They may not be in this room, but they are from this local area. I do not know which people you will make decisions with because you will be mixed with lots of other people in order to make a group of four. All of the people you are mixed with are from around here.

At this point the local helper should begin passing out the materials (including the example sheet for Decision 1). This material should all be bundled together, except for the comprehension sheet, to make it easy to pass out.

I am now going to pass out your materials. You should have an envelope marked "Personal" and an envelope marked <*Local*>. You should have 10 red tokens (each of which are worth <$.50> to you and everyone else). Your tokens are in your "Personal" envelope. Please take them out and count them to make certain you have 10.

The first thing I would like you to do is take two stickers off your ID card and put it on the upper right corner of both your envelopes. Please make certain you do this. This is the only way we can make certain you will be paid.

At this point the experimenter can demonstrate how this is done on a blank envelope.

Also, it is important that you do not write on, fold, or damage the envelopes in any way. Only your ID sticker should be on the envelope.

Before you make your decision, I want to make certain you understand how you get paid. Please make certain you know exactly how you can receive money. You will be paid based on the decisions that you and the others you are mixed with make.

Once I am finished with the examples you will make your own decision about how many red tokens you will put in your "Personal" envelope and how many tokens you will put in your <*Local*> envelope.

Please follow along with the examples that have been handed out to you. For example #1, suppose that you put 10 red tokens in your "Personal" envelope and the other three people put a total of 12 red tokens in their <Local> envelopes. In that case, the 12 tokens in the local pot will be doubled (to 24) and shared equally among you and the other three people (6 each).

Example 1

You put 10 red tokens in your "Personal" envelope. Others put a total of 12 red tokens in their <Local> envelopes. Those <Local> envelope tokens are doubled and you get an equal share.

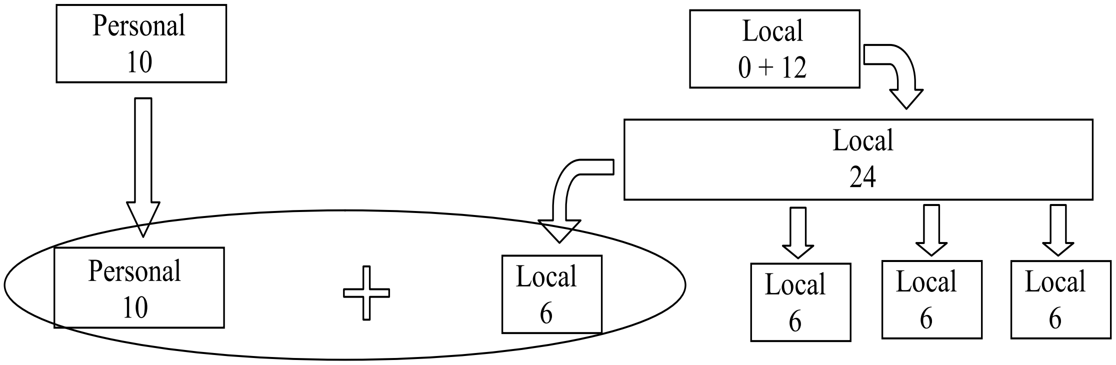

You would then receive a total of 16 tokens: 10 from your personal envelope that you kept, and 6 from your share of the local pot; given that there were 12 red tokens in the <Local> envelopes,

that amount would be doubled to 24 by me and you would get an equal share, which is 6 red tokens. You would end up with 16 tokens worth <$8.00>. Is anyone uncertain about how this happens?

To take another simple example (#2) suppose you put 8 of your red tokens in the <Local> envelope and no one else put any red tokens in the <Local> envelope. What would you receive? If you like you can write in the blanks on the example.

Example 2

You put 2 red tokens in your "Personal" envelope. Others put 0 red tokens in their <Local> envelopes.

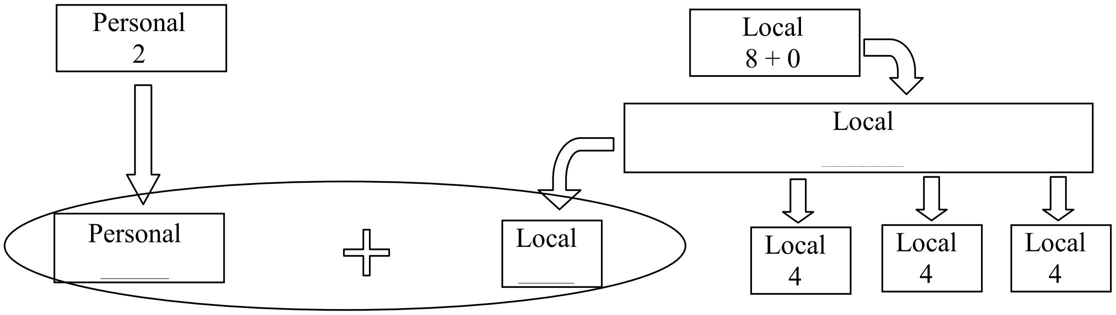

Wait while participants make calculations; look around to see if they are attempting to come up with the answer; encourage someone to give an answer.

You would receive a total of 6 tokens. First you would have 2 red tokens in your "Personal" envelope. Given that there were 8 red tokens in the <Local> envelopes (all put there by you) that amount would be doubled to 16 and you would get an equal share, which is 4 red tokens. The other people in your local group also get 4 red tokens. You would end up with 6 tokens worth <$3.00>.

Finally, let me give one more example (#3). Suppose you put all 10 of your red tokens in the <Local> envelope. Suppose that the other 3 people did the same thing. That means a total of 40 red tokens in the <Local> envelopes. How much would you receive?

Example 3

You put 0 red tokens in your "Personal" envelope. Others put 30 red tokens in their <Local> envelopes.

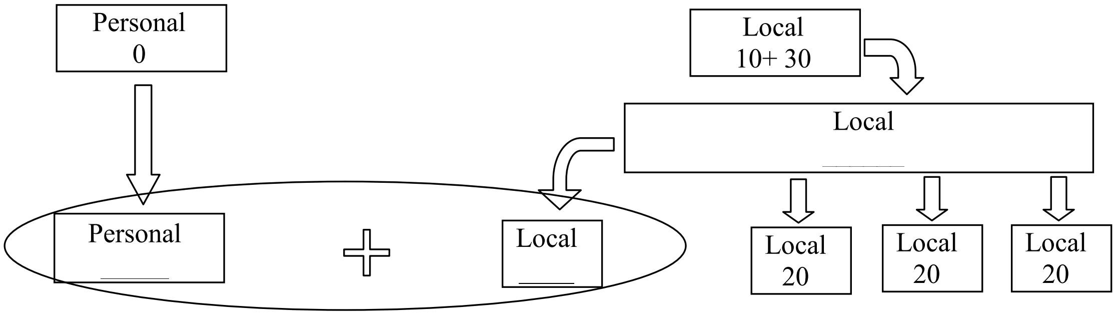

Wait for an answer from participants…

You (and the other three people) would receive a total of 20 tokens, which is <$10.00> for this decision. First you would have 0 red tokens in your "Personal" envelope. Second, in the <Local> envelopes there would be 40 tokens. This would be doubled to 80 and your share would be 20 tokens.

These three examples show that what you get can be very different, depending both on what you and everyone else does. Please take the time to look through the examples. Do this while we are passing out a new sheet of paper.

At this point pass out the comprehension sheet.

A sheet is being handed out to you with three questions on it. Please do not answer the questions until I read them aloud.

When the sheets are handed out, begin the instructions.

Before you do anything, please remove one of your stickers and put it in the upper right hand corner of the sheet that was just handed out.

Pause until everyone has done so.

I am going to read the questions one at a time. Please check the answer you think is most appropriate. When everyone is done I will read the answers.

1. What happens when a red token is put into the <Local> envelope? (Nothing; The token is cut in half; The token is doubled; The token is tripled)

2. How many people, including you, are in the <Local> group? (Two people; Three people; Four people; Five people)

3. Everyone gets an equal share of the <Local> envelope. (True or False?)

Pause until everyone is finished. These sheets will be collected with the envelopes.

Now that you are done, I will read the answers. Do not mark your papers. They will be collected later.

1. What happens when a red token is put into the <Local> envelope? The red token is doubled and your get an equal share along with the other people in the <Local> group.

2. How many people, including you, are in the <Local> group? There are a total of four people in the local group. This includes you. This means each of you will get a one-quarter share of the red tokens that are put into the local envelopes and doubled.

3. Everyone gets an equal share of the <Local> envelope. This is true – everyone gets an equal share.

Now it is time for you to make your decision. You can put any combination of tokens into the 2 envelopes. Remember that the red tokens you put into your "Personal" envelope are yours and will not be divided among any others. Whatever you and the three other people from around this area put into the <Local> envelopes will be doubled. Each of you will get an equal share of that amount. Please make your decision and then place the envelopes on your [box/desk]. DO NOT seal the envelopes. My assistant will come around and collect your envelopes and all your materials. The assistant will check to make certain you have put your ID number in the upper right corner of your envelopes. When you have finished put your envelopes on top of your [box/desk] so we will know you are finished. If you have any questions please raise your hand.

The envelopes will be put into a box marked Decision 1. The assistant should double check each envelope to make certain that it has an ID number attached to it. The comprehension sheet should also have an ID on it. If not, ask the subject to do it before the envelopes are placed in the box. Also collect any other materials from the subjects.

The envelopes should be taken to a Core member of the team who is outside the room. The Core member should open the personal envelopes and enter and record the number of red tokens for each subject. The Core member should then open the <local> envelope, enter and record the number of red tokens, check the group assignment and calculate the share obtained by the S. The personal tokens and the Ss group share should be filled out on the decision record slip of paper for each participant.

The data for each participant's choices and payment should also be entered onto the session spreadsheet.

Now that everyone's decision has been made, the envelopes will be matched with other people and how much money you receive will be calculated. It will take a while to do this. At the end of the session you will be given an envelope with your payment.

DECISION TWO.

You have now finished the first decision. The second decision is slightly different, so please listen very carefully. In this decision you will have 10 blue tokens and 3 envelopes. Once again you will be paid <$.50> for each blue (colored) token.

In this decision you will be randomly mixed with different groups of people. The first group will be similar to the first decision. You will be mixed with three other people from this local area. It is very likely that this will be three different people than the first time. The second group will be composed of 12 people. It will include the three local people, plus two other groups of four people from other areas in this country.

As with the first decision, the blue tokens you put in your "Personal" envelope will be yours and not divided with anyone else. Second the blue tokens you and the others put into the <Local> envelope will be doubled and you will get a 1/4 share from the local group. Finally you have a <COUNTRY NAME> envelope. The blue tokens that all 12 people put into those envelopes will be tripled. You will get an equal share of the tripled amount.

> *The assistant hands out the bundle of materials (including the example sheet for Decision 2); Ss each get a bundle.*

Your task is to put 10 tokens in the envelopes. You can put them in any combination that you please. My assistant will now hand out these materials. You should get 10 blue tokens and 3 envelopes. You should have 10 blue tokens (each of which are worth <$.50> to you and everyone else). Your tokens are in your "Personal" envelope. Please take them out and count them to make certain you have 10.

Please remove an ID sticker and put one on each of the three envelopes. Please do this now and make certain it is in the upper right corner.

Please follow along with the examples that have been handed out to you. For example 1 suppose you put 10 blue tokens in your “Personal” envelope, others put 10 blue tokens into their <Local> envelopes and 12 blue tokens were put into the <COUNTRY NAME> envelopes. How much would you receive?

Example 1

You put 10 blue tokens in your “Personal” envelope. Others put a total of 10 blue tokens in their <Local> envelopes. Finally, 12 blue tokens are put in the “US” envelopes. Those “US” envelope tokens are tripled and you get an equal share.

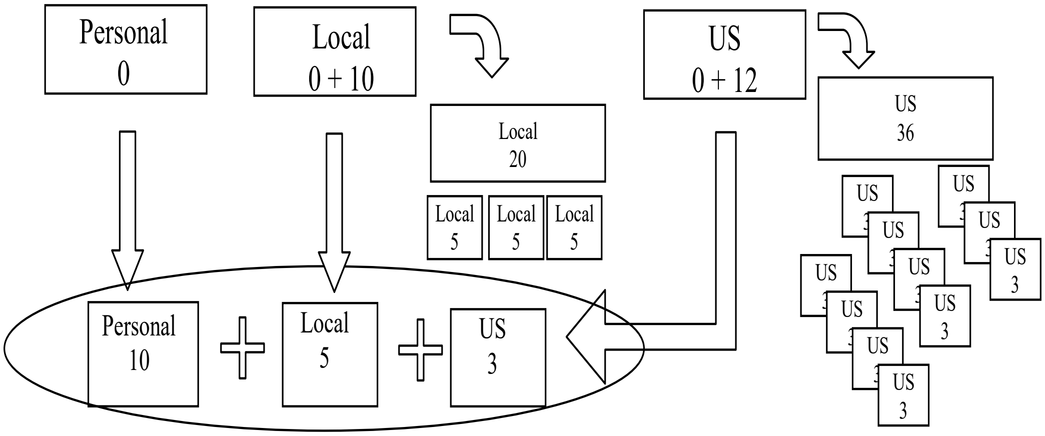

This is a little complicated, but is easy to figure out. First you would get 10 tokens from your “Personal” envelope. Second you would get 5 tokens from the <Local> share (the 10 tokens would be doubled and your share is 5). Finally you would get 3 tokens from the <COUNTRY NAME> share (the 12 tokens would be tripled to 36 and divided by 12, you get 3). Your total is 18 blue tokens and for that you would receive <$9.00≥. Note that every other member of your local group would get 5 tokens from the local share and 3 from their share of the <COUNTRY NAME> one, but the total tokens received by each of them would depend on the number of tokens allocated to the Personal envelope.

Here’s another example (#2). Suppose you put your 10 blue tokens in the <COUNTRY NAME> envelope and 2 other tokens were put into the <COUNTRY NAME> envelope. No tokens were

put into the local envelopes. How much would you receive? Go ahead and write on your example if you would like.

Example 2

You put 0 blue tokens in your “Personal” envelope. Others put 0 blue tokens in their <Local> envelopes. Finally, 12 blue tokens are put in the “US” envelopes.

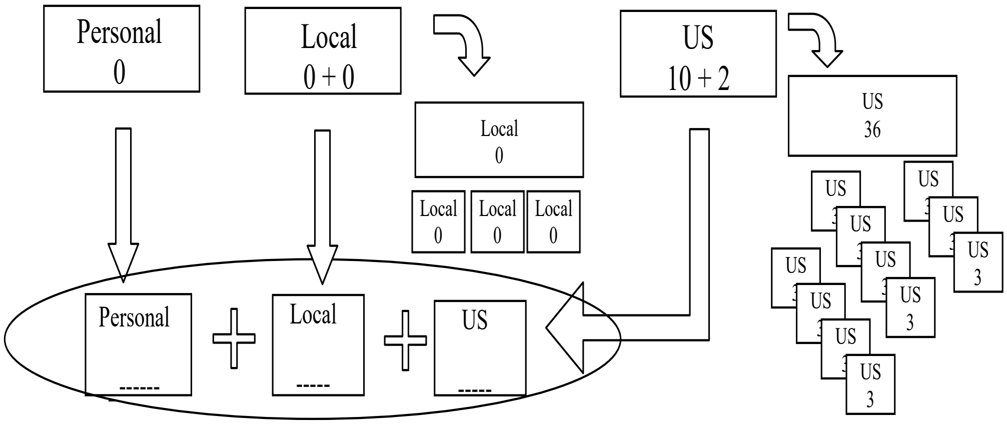

Pause and wait for participant to come up with an answer…

You would get nothing from your “Personal” envelope. Because no one else put anything into their <Local> envelopes you would get no share from that. Finally, the 12 blue tokens in the <COUNTRY NAME> envelopes would be tripled and your share would be 3 tokens. You would receive <$1.50>.

In the final example (#3) suppose you put no blue tokens in your “Personal” envelope, you put 2 tokens in your local envelope and the other three people put 10 blue tokens in their <Local> envelopes for a total of 12, and you and the other 11 people put a total of 80 blue tokens into their <COUNTRY NAME> envelopes. How much would you receive?

Example 3

You put 0 blue tokens in your "Personal" envelope. You and the others put a total of 12 blue tokens in their <Local> envelopes. Finally, 80 blue tokens are put in the "US" envelopes.

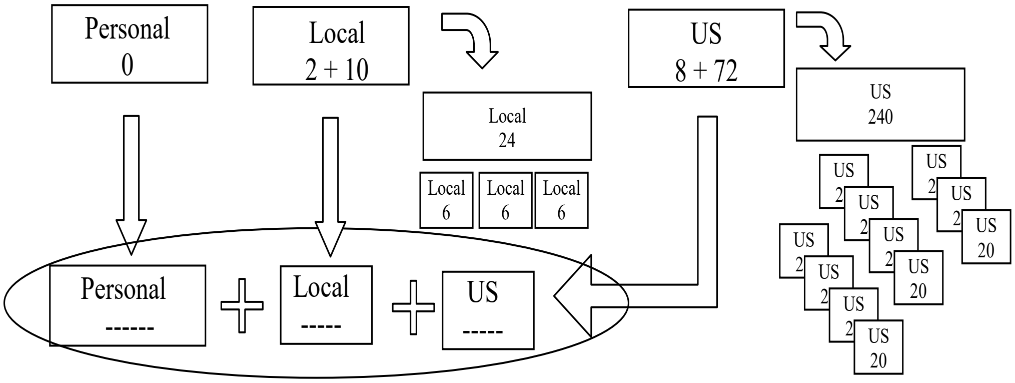

Wait for participant to come up with an answer…

Once again you would get nothing from your "Personal" envelope. The 12 tokens in the local envelopes would be doubled and your share (1/4) would be 6. In the <COUNTRY NAME> envelopes there are 80 blue tokens. These are tripled to 240 and your share (1/12th) is 20 blue tokens. For this you would receive a total of 26 tokens, worth <$13.00>. Again, does everyone understand how this result was obtained? These three examples show that what you get depends both on what you and everyone else does. Please take a minute to go through the examples.

Pause for one minute while people look over their materials.

Now it is time for you to make your decision. You can put any combination of tokens into the 3 envelopes. Remember that the blue tokens you put into your "Personal" envelope are yours and will not be divided among any others. Whatever you and the three other people from around this area put into the <Local> envelopes will be doubled. Each of you will get an equal share of that amount. Whatever you put into the <COUNTRY NAME> envelope will be tripled. You and 11 others will get an equal share of that amount. Please make your decision, DO NOT seal the envelopes and put the materials on top of your [desk/box]. My assistant will come around and collect your envelopes. The assistant will check to make certain you have put your ID number in the upper right corner of all three of your envelopes. When you are finished, please put all of your materials on top of your [box/desk].

The envelopes will be put into a box marked Decision 2. The assistant should double check each envelope to make certain that it has an ID number attached to it. If not, ask the subject to do it before the envelopes are placed in the box. Collect all materials.

The envelopes should be taken to a Core member of the team who is outside the room. The Core member should open the personal envelopes, enter and record the personal decision for each subject. The Core member should then open the local envelope, enter and record the number of blue tokens, check the group assignment, and calculate the share obtained by the Ss. The group total and the Ss share should be filled out on the decision slip of paper and the session log. Finally the <COUNTRY NAME> envelope should be opened, the appropriate information recorded and group totals calculated under the matching routine. The group total and the Ss share should be filled out on the decision slip and the session log.

Now that everyone's decision has been made, the envelopes will be matched with other people and how much you money you receive will be calculated. It will take a while to do this. At the end of the session you will be given your payment in an envelope.

DECISION THREE.

You have now finished the second decision. The third decision is similar, but it has some changes, so please listen very carefully. In this decision you will have 10 yellow tokens and 3 envelopes. Once again you will be paid <$.50> for each yellow (colored) token.

In this decision you will be randomly mixed with different groups of people. First you will be mixed with three other people from this local area. It is very likely that it will be three people who are different from your first or second decision. The second group will be composed of 12 people. It will include the same three local people, plus two other groups of four people from countries around the world.

As I told you at the beginning, this research is being conducted by an international team. This study is being conducted with participants from all parts of the world, including Asia, Africa, Europe, and North and South America. The team is collecting decisions made by other people who are facing the same choices as you, and they are sending their results back to a central administrator who will tell us what different groups have decided to do. Your decision will be randomly mixed with what other people have done and this will determine your final payment. Your decisions will also affect the payments of the others in your group.

As with the second decision, the yellow tokens you put in your “Personal” envelope will be yours and not divided with anyone else. Second the yellow tokens you and the others put into the <Local> envelope will be doubled and you will get a 1/4 share from the local group. Finally you have a <WORLD> envelope. The yellow tokens that you and 11 other people put into those envelopes will be tripled. You will get an equal share of the tripled amount. The materials that my assistant will now hand out are similar to what you received for the previous decision. Please look these over and be sure that you understand before you begin.

Hand out Decision 3 materials.

Your task is to put your 10 tokens in the envelopes. You can put any combination of tokens into the 3 envelopes. You should have 10 yellow tokens (each of which are worth <$.50> to you and everyone else). Your tokens are in your “Personal” envelope. Please take them out and count them to make certain you have 10.

Please remove an ID sticker and put one on each of the three envelopes. Please do this now and make certain it is in the upper right corner.

Now it is time for you to make your decision. Remember that the yellow tokens you put into your "Personal" envelope are yours and will not be divided among any others. Whatever you and the three other people from around this area put into the <Local> envelopes will be doubled. Each of you will get an equal share of that amount. Whatever you put into the <WORLD> envelope will be tripled. You and 11 others will get an equal share of that amount. Please make your decision, DO NOT seal the envelopes and put the materials on top of your [desk/box]. My assistant will come around and collect your envelopes. The assistant will check to make certain you have put your ID number in the upper right corner of all three of your envelopes. When you are finished, please place all your materials on top of your [box/desk].

The envelopes will be put into a yellow box marked Decision 3. The assistant should double check each envelope to make certain that it has an ID number attached to it. If not, ask the subject to do it before the envelopes are placed in the box. Collect all materials.

The envelopes should be taken to a Core member of the team who is outside the room. The Core member should open the personal envelopes, enter and record the personal decision for each subject. The Core member should then open the local envelope, enter and record the number of yellow tokens; check the group assignment and calculate the share obtained by the S. The group total and the Ss share should be filled out on the decision slip and the session log sheet. Finally the world envelope should be opened, the appropriate information entered and recorded and group totals calculated under the matching routine. The group total and the Ss share should be filled out on the decision slip and the session log.

The core member then combines information from all three decisions for each participant and calculates their total payment, which is recorded on the decision slip for that participant (and on the session log sheet). The decision slip is put into an envelope, along with the total payment in money (rounded to the nearest whole number) and labeled with the participant ID number.

You have now finished making your decisions with others. Before you are given the payments from your decisions, I am going to have my assistant pass out a questionnaire. This questionnaire will help us get more information about the people who participated in the decisions. Your questionnaire will have only your ID number. We will not know who you are and how you responded. Please be as honest as you can with your answers.

The assistant passes out the questionnaire, with the expectancy measures first.

Please take the sticker off of your ID card and put it in the upper right corner of the first page of the questionnaire you have been handed. When you are finished, please place your questionnaire and pen on top of your [box/desk]. Once everyone is finished, we will wait while the payment calculations are being completed.

When all participants are finished they can be brought out one at a time, with their questionnaire, The questionnaire can serve as the "passport" out of the experiment;tt needs to have an ID on it for the subject to collect a pay envelope. The experimenter will then give the participant the payment envelope which matches the participant's ID#.

Participants should then count and verify that they have received the amount listed on the decision slip which is in the envelope, and the experimenter will fill in the participant's ID number on the log sheet, the amount received, and then put his/her initials next to it. The experimenter will then cover that particular line of the log sheet and the next participant will come in.

Decision 1

Example 1

You put 10 red tokens in your "Personal" envelope. Others put a total of 12 red tokens in their <Local> envelopes. Those <Local> envelope tokens are doubled and you get an equal share.

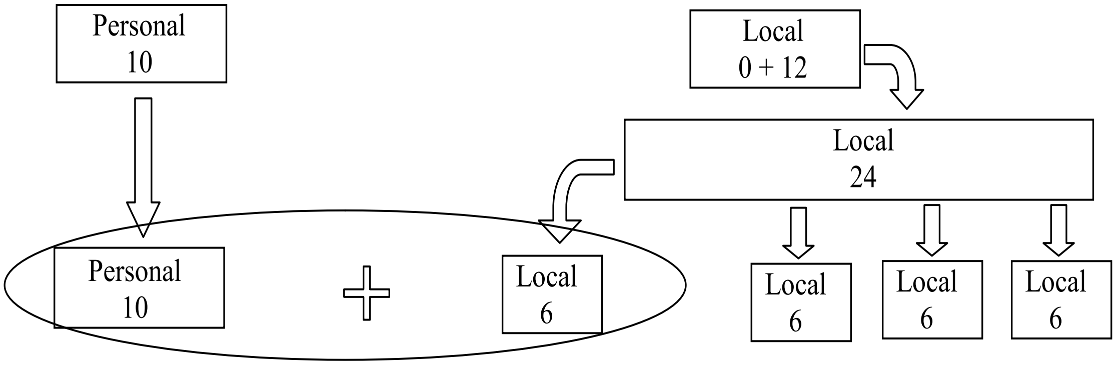

Example 2

You put 2 red tokens in your "Personal" envelope. Others put 0 red tokens in their <Local> envelopes.

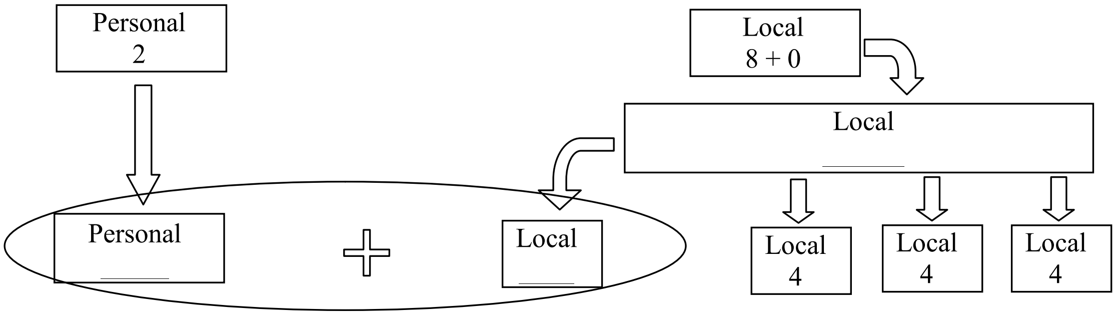

Example 3

You put 0 red tokens in your "Personal" envelope. Others put 30 red tokens in their <Local> envelopes.

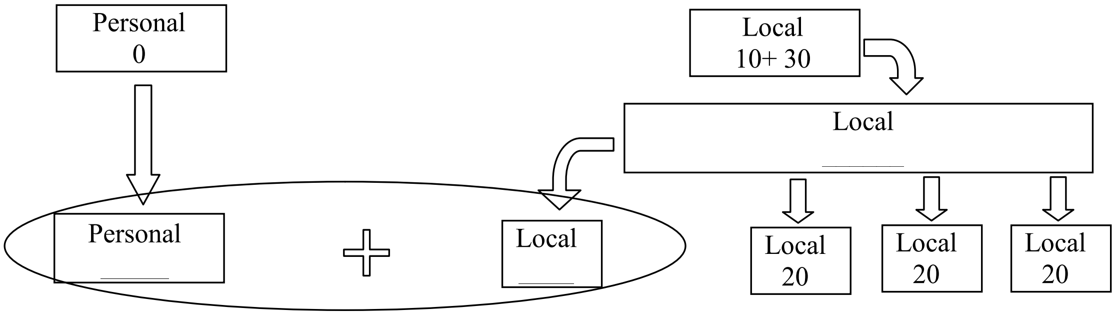

ID

Decision 1

Please check the correct box for each question.

1. What happens when a red token is put into the <Local> envelope?

- θ **Nothing**
- θ **The token is cut in half**
- θ **The token is doubled**
- θ **The token is tripled**

2. How many people, including you, are in the <Local> group?

- θ **Two people**
- θ **Three people**
- θ **Four people**
- θ **Five people**

3. Everyone gets an equal share of the <Local> envelope.

- θ **True**
- θ **False**

Decision 2

Example 1

You put 10 blue tokens in your “Personal” envelope. Others put a total of 10 blue tokens in their <Local> envelopes. Finally, 12 blue tokens are put in the “US” envelopes. Those “US” envelope tokens are tripled and you get an equal share.

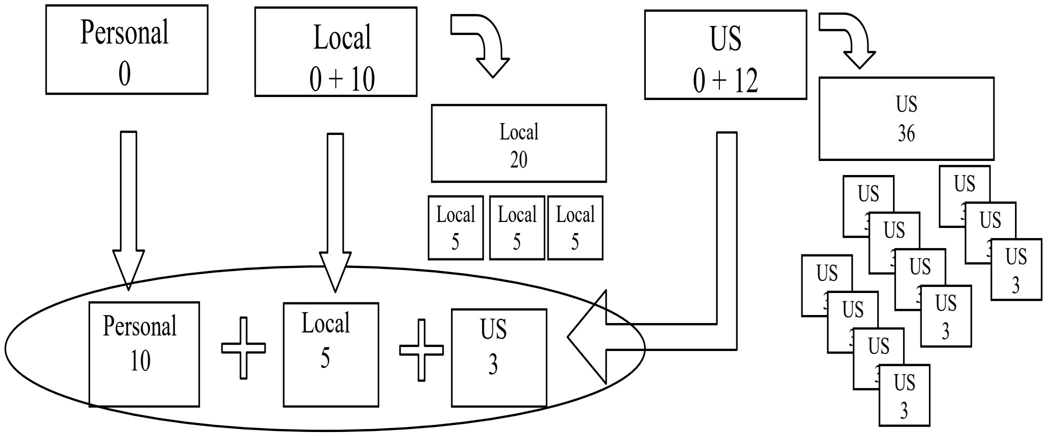

Example 2

You put 0 blue tokens in your “Personal” envelope. Others put 0 blue tokens in their <Local> envelopes. Finally, 12 blue tokens are put in the “US” envelopes.

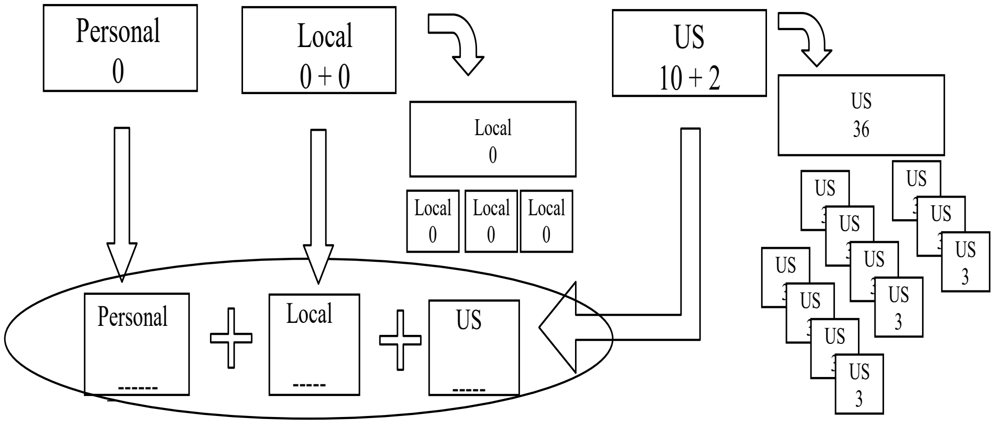

Example 3

You put 0 blue tokens in your "Personal" envelope. You and the others put a total of 12 blue tokens in their <Local> envelopes. Finally, 80 blue tokens are put in the "US" envelopes.

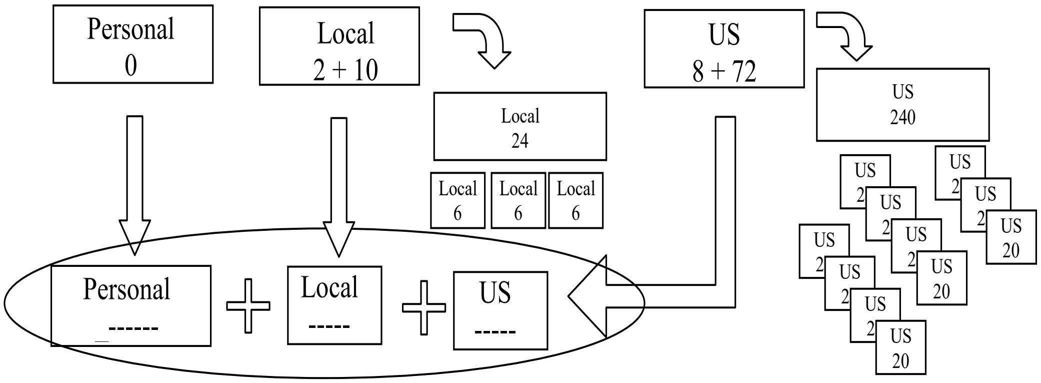

ID

Decision 1		
	Earnings from Decision 2	______________
Decision 2		
	Earnings from Decision 2	______________
Decision 3		
	Earnings from Decision 3	______________
Total Experiment Earnings in this Envelope	(rounded)	______________
Show-up Fee		______________
Total Amount of Money Claimed by Participant	(rounded)	______________

ID

In Decision 1 you had 10 red tokens. So did everyone else. You could put your tokens into your “Personal” envelope or into your <Local> envelope. The other three people in your local group could also choose to put tokens into their own personal envelope or into the <Local> envelope. Please answer the following questions.

1. How much do you think the other three people put into the <Local> envelopes in total (there is a maximum of 30 red tokens that they could put into them): _______________

2. How much money do you expect to receive from the <Local> envelope that will be returned to you: ____________

3. How much did you feel you were obliged to put money in the <Local> envelope, no matter what other people in the group did? (check one response)

____Not at all obliged

___ Somewhat obliged

__ Strongly obliged

In Decision 2 you had 10 blue tokens. So did everyone else. You could put your tokens into your "Personal" envelope, into your <Local> envelope or into the *<COUNTRY NAME>* envelope. Please answer the following questions.<br><br>1. How much do you think the other three people in your local group put into their <Local> envelopes (a maximum of 30 blue tokens that could be put into them): _______________<br><br>2. How much do you think the other 11 people in your *<COUNTRY NAME>* put into the "US" envelopes in total (a maximum of 110 blue tokens could be put into them): __________<br><br>3. How much did you feel you were obliged to put money in the *<COUNTRY NAME>*envelope, no matter what other people in the group did? (check one response)<br><br>___Not at all obliged<br><br>___Somewhat obliged<br><br>__Strongly obliged

In Decision 3 you had 10 yellow tokens. So did everyone else. You could put your tokens into your "Personal" envelope, into your <Local> envelope or into the "World" envelope. Please answer the following questions.

1. How much do you think was put into the <Local> envelopes by the other three people in your local group (a maximum of 30 yellow tokens that could be put into them): _______________

2. How much do you think was put into all of the "World" envelopes by the other 11 people in your group (a maximum of 110 yellow tokens could be put into them): __________

3. How much did you feel you were obliged to put money in the WORLD envelope, no matter what other people in the group did? (check one response)

___Not at all obliged

___Somewhat obliged

__Strongly obliged

SM.4: Research questionnaire

Note: Terms in italics were adapted to different localities / countries. The terms reported below were used in the community of Columbus, OH, US. The numbering of the question is the same as in the original questionnaire.

{Classification Codes: [S]=Social globalisation; [C] = Cultural globalisation; [E]=Economic globalization; [GA]= Global Awareness; [SI]=Social Identity; [AMI]=Association Membership Index; [GAMI]=Global Association Membership Index }

1. How often do you normally use the following products or services? Check one option.

		I own/have access to this product or service, and I use it:				I do not own/have access to this product or service.
		Every day	Every week	Less often	Never	
**[S]**	Internet	☐1	☐2	☐3	☐4	☐5
**[ECO]**	Credit card	☐1	☐2	☐3	☐4	☐5

2. If you use the following products or services, do you use them to contact people living in other parts of your country, or people living in other countries? Check all that apply.

		Local area	Other parts of my country	Other countries	Does not apply
**[S]**	a. Landline phone	☐1	☐2	☐3	☐4
**[S]**	b. Mobile phone	☐1	☐2	☐3	☐4
**[S]**	c. Email	☐1	☐2	☐3	☐4
**[S]**	d. Postal mail	☐1	☐2	☐3	☐4
**[E]**	e. Fax machine	☐1	☐2	☐3	☐4

3. Consider the following geographical areas. How often do you travel, either for work or for vacation, in each of them? Check one.

		Every week	Every month	Every year	Less often	Never
	a. Within a national area (to other parts of my country besides my own locality).	☐1	☐2	☐3	☐4	☐5
**[C]**	b. To other countries within my continent	☐1	☐2	☐3	☐4	☐5
**[C]**	c. To other countries outside my continent	☐1	☐2	☐3	☐4	☐5

4. How concerned are you with the following issues? Check one.

		Not at all concerned	Slightly concerned	Concerned	Very concerned	I am not informed about this issue.
**[GA]**	a. Global warming	☐1	☐2	☐3	☐4	☐5
**[GA]**	b. The spread across the planet of potentially dangerous diseases (for example, HIV, SARS, bird flu)	☐1	☐2	☐3	☐4	☐5
**[GA]**	c. Making the action of International Criminal Courts of justice more effective	☐1	☐2	☐3	☐4	☐5
**[GA]**	d. The persistent gap between rich and poor people around the world	☐1	☐2	☐3	☐4	☐5

5. Have you taken part in the following activity?

		Yes	No
	a. Contributing to international aid efforts for natural disasters (for example, hurricanes in the US, earthquakes in Pakistan, tsunami)	☐1	☐2
	b. Contributing or participating in international aid efforts for poverty relief (for example, Live Eight)	☐1	☐2
**[C]**	c. Following international sport events (for example, Olympic games, soccer world cup)	☐1	☐2
**[C]**	d. Following international cultural events or international trade fairs	☐1	☐2

6. How often do you do the following activity? Check one.

		I own/have access to this product or service, and I use it:				I do not own/have access to this product or service.
		Every day	Every week	Less often	Never	
**[C]**	a. Watch a television program or a movie from a different country	☐1	☐2	☐3	☐4	☐5
**[C]**	b. Watch / listen to an international news source (CNN International, BBC World, *Euronews*)	☐1	☐2	☐3	☐4	☐5
**[C]**	c. Read an international news source (Time, The Economist, *Le Monde*)	☐1	☐2	☐3	☐4	☐5
**[C]**	d. Read an international magazine other than a news related publication, e.g. a style or sports magazine (Cosmopolitan, National Geographic, *Men's Health*)	☐1	☐2	☐3	☐4	☐5
**[C]**	e. Read a book written by an author from another country	☐1	☐2	☐3	☐4	☐5
**[C]**	f. Listen to music made by artists from other countries	☐1	☐2	☐3	☐4	☐5

9. **[E]** Do you work for a multinational or foreign-owned company?

Yes	No
☐1	☐2

10. **[E]** If you (or your household) own a car, where was it made? Check one (if you own more than one car, consider the one that you regularly use).

In my country	In a different country	I do not know where my car was made	I do not own a car
☐1	☐2	☐3	☐4

11a. Consider the following list; Are products or services that are from different parts of the world available in the area where you live?

	Yes	No	I don't know
A. Restaurants (e.g. *Japanese, Thai* restaurants)	☐1	☐2	☐3
B. Food and beverages (from supermarkets, shops or bars)	☐1	☐2	☐3
C. Clothing	☐1	☐2	☐3

11b. **[E]** If you have answered yes to the previous question, how often do you use such products or services?

	Every day	Every week	Less often	Never
a. Restaurants (e.g. *Japanese, Thai* restaurants)	☐1	☐2	☐3	☐4
b. Food and beverages (from supermarkets, shops or bars)	☐1	☐2	☐3	☐4
c. Clothing	☐1	☐2	☐3	☐4

12a. Consider the following list; Are products or services that are produced by multi-national companies - that is, those companies active in different parts of the world - available in the area where you live?

	Yes	No	I don't know.
A. Restaurants and cafes (e.g. Mc Donald's, Starbucks Coffee, *Pizza Hut, Taco Bell*)	☐1	☐2	☐3
B. Food and Beverages (e.g. Coca-Cola, Nestlé, *Dannon*)	☐1	☐2	☐3
C. Clothing (e.g. Nike, Zara, *Adidas, Levi's*)	☐1	☐2	☐3

12b. **[E]** If you have answered yes to the previous question, how often do you use such products?

	Every day	Every week	Less often	Never
A. Restaurants and cafes (e.g. Mc Donald's, Starbucks Coffee, *Pizza Hut, Taco Bell*)	☐1	☐2	☐3	☐4
B. Food and Beverages (e.g. Coca-Cola, *Nestle', Dannon*)	☐1	☐2	☐3	☐4
C. Clothing (e.g. Nike, Zara, *Adidas, Levi's*)	☐1	☐2	☐3	☐4

14. Currently do you own any of the following?

		Yes	No
**[E]**	a. Foreign currencies	☐1	☐2
**[E]**	b. Bank deposit in another country	☐1	☐2
**[E]**	c. Some investment(s) in another country	☐1	☐2

15. **[S]** Besides your native tongue, how many other languages can you speak?

None	☐1
I can understand and can make myself understood in another language.	☐2
I am fluent in another language.	☐3
I am fluent in more than one other language.	☐4

16a. How many different immigrant communities live in the area where you live (for example, *Hmong immigrants*)?

None	☐1
Between 1 and 2	☐2
Between 3 and 4	☐3
More than that	☐4

17a. How many ethnic/racial groups different from yours live in the area where you live (for example, *White, Black/African Americans, Asian, Hispanic*)?

None	☐1
Between 1 and 2	☐2
Between 3 and 4	☐3
More than that	☐4
Several people (6 or more people)	☐3

21. **[SI]** How strongly do you feel attachment to your community in *Columbus*, in the *United States*, or to the world as a whole?

	Not attached at all			Very attached
a. Your local community	☐1	☐2	☐3	☐4
b. Your country	☐1	☐2	☐3	☐4
c. The world as a whole	☐1	☐2	☐3	☐4

22. **[SI]** How strongly do you define yourself as a member of your community in *Columbus*, in the *United States*, or of the world as a whole?

	Not at all			Very strongly
a. Your local community	☐1	☐2	☐3	☐4
b. Your country	☐1	☐2	☐3	☐4
c. The world as a whole	☐1	☐2	☐3	☐4

23. **[SI]** How close do you feel to other members of your community in *Columbus*, in the *United States*, or to the world as a whole?

	Not at all close			Very Close
a. Your local community	☐1	☐2	☐3	☐4
b. Your country	☐1	☐2	☐3	☐4
c. The world as a whole	☐1	☐2	☐3	☐4

25. **[AMI]** Please look carefully at the following list of voluntary organizations and activities. How would you describe your involvement with them? Check the one response that best applies for each type of activity or organization.

	I do not belong and do not follow their activities.	I do not belong but I sympathize with some of their activities.	I Belong
a. Social welfare services for elderly, *handicapped* or deprived people	☐1	☐2	☐3
b. Religious or church organizations	☐1	☐2	☐3
c. Education, arts, music or cultural activities	☐1	☐2	☐3
d. Labor unions	☐1	☐2	☐3
e. Political parties or groups	☐1	☐2	☐3
f. Poor countries development or human rights	☐1	☐2	☐3
g. Conservation, environmental, animal rights groups	☐1	☐2	☐3
h. Professional associations	☐1	☐2	☐3
i. Youth work (for example, *scouts, guides, youth clubs*, etc.)	☐1	☐2	☐3
j. Sports or recreation	☐1	☐2	☐3

k. Women's group	☐1	☐2	☐3
l. Peace movement	☐1	☐2	☐3
m. Voluntary organizations connected with health	☐1	☐2	☐3

26. **[GAMI]** If you have answered that you belong to some of the organizations listed above, please indicate whether the voluntary activity or organization is mainly active locally, nationally or internationally. Check as many responses as apply for each type of activity or organization.

	Local	National	International
a. Social welfare services for elderly, handicapped or deprived people	☐1	☐2	☐3
b. Religious or church organizations	☐1	☐2	☐3
c. Education, arts, music or cultural activities	☐1	☐2	☐3
d. Labor unions	☐1	☐2	☐3
e. Political parties or groups	☐1	☐2	☐3
f. Poor countries development or human rights	☐1	☐2	☐3
g. Conservation, environmental, animal rights groups	☐1	☐2	☐3

h. Professional associations	☐1	☐2	☐3
i. Youth work (for example, scouts, guides, youth clubs, etc.)	☐1	☐2	☐3
j. Sports or recreation	☐1	☐2	☐3
k. Women's group	☐1	☐2	☐3
l. Peace movement	☐1	☐2	☐3
m. Voluntary organizations connected with health	☐1	☐2	☐3

27. For each of the following statements, please state if you agree or disagree:

a. Our people are not perfect, but our culture is better than all others.

Completely disagree	Somewhat disagree	Somewhat agree	Completely agree
☐1	☐2	☐3	☐4

b. Our way of life needs to be protected against foreign influence.

Completely disagree	Somewhat disagree	Somewhat agree	Completely agree
☐1	☐2	☐3	☐4

c. We should restrict and control entry of people into our own country more than we do.

Completely disagree	Somewhat disagree	Somewhat agree	Completely agree
☐1	☐2	☐3	☐4

28. What do you think about each of the following: Has it been a very good thing, somewhat good, somewhat bad or very bad for you (and your family)?

28a. The world becoming more connected through greater economic trade and business ties?

Very Good	Somewhat Good	Somewhat Bad	Very Bad	Don't Know
☐1	☐2	☐3	☐4	☐5

28b. The world becoming more connected through faster communication and greater movements of people?

Very Good	Somewhat Good	Somewhat Bad	Very Bad	Don't Know
☐1	☐2	☐3	☐4	☐5

30. What is your sex?

Male	Female
☐1	☐2

31. In which year were you born? ____________________

32. What is the highest level of education you completed?

Grade School	*High School*	*Technical School*	*Bachelors Degree*	*Masters Degree*	*Doctoral Degree*
☐1	☐2	☐3	☐4	☐5	☐6

			Yes	No
**[S]**	33a.	Were you born in a country different than the US?	☐1	☐2
**[S]**	33b.	Were any of your parents born in a country different than *the US*?	☐1	☐2

35. What is your marital status?

Single	Married	Divorced/ Separated	Widowed	Living with partner
☐1	☐2	☐3	☐4	☐5

36. What is your current employment situation?

Full-time employed	☐1
Part-time employed	☐2
Self-employed	☐3
Retired/Pensioned	☐4
Housewife/husband not otherwise employed	☐5
Student	☐6
Unemployed	☐7
Other (Please specify) ________________________	☐8

37. In which profession/occupation do you or did you work? If more than one job, the main job? What is/was your job there?

____

38. Here is a scale of incomes. We would like to know in what group your household is, counting all wages, salaries, pensions and other incomes that come in. Just check the group your household falls into, before taxes and other deductions.

$0-9,999	*10,000-14,999*	*15,000-24,999*	*25,000-34,999*	*35,000-49,999*	*50,000-74,999*	*75,000-99,999*	*100,000-149,999*	*150,000-199,999*	*Over 200,000*
☐1	☐2	☐3	☐4	☐5	☐6	☐7	☐8	☐9	☐10

Notes to Local Collaborators for Adapting Questionnaire to Local Environment

The present questionnaire is the version that was tailored for a US location (Portage, WI) for a pilot test. Hence, several questions require adaptation to the country/locality where the research is conducted. In particular, all items highlighted in yellow require some change. Some of these are obvious, e.g. substituting the name of your country for the US. Some others are less obvious. In particular, this is the case for the questions that provide examples for certain items, such as satellite channels, newspapers, *etc*. Normally, there will be 3 examples for each of these items, which include (a) the most widespread item worldwide; (b) the most widespread item worldwide coming from a different continent than the first one (this is to avoid listing mainly made-in-US brands); (c) another widespread item in the country's region. By region we mean the continental or sub-continental geographical entity to which a country belongs (e.g. North-America; Latin America; Sub-Saharan Africa; Europe; Asia; former Soviet Republics; Middle East). The questionnaire generally provides examples for (a) and (b), but identifying (c) is left to the local researcher's expertise. The following are suggestions derived from a variety of sources (PEW Global Attitudes Project: 06.03.03, Views of a Changing World, Summer 2002 44-Nation Survey http://pewglobal.org/datasets/, UNCTAD databases, internet):

Q6b: ask "CNN International, BBC World" in every country. Add a third example in each country as per Q60 in Pew survey, or local researcher's advice (e.g. Al Jazeera for Iran; Euronews for Russia and Europe; another region-wide channel for the US; DSTV for South Africa).

Q6c: Ask "Time, the Economist" in every country; provide a third example as per local researcher's advice (e.g. International Herald Tribune for non-English speaking countries; Le Monde for English-speaking countries).

Q6d: Ask "Cosmpolitan, National Geographic" in every country. Then ask another magazine widespread in the region (e.g. Men's Health, or Reader's Digest). You can find other examples at http://www.allyoucanread.com/Top20/ (Note: ignore adult magazines).

Q11a,b: provide example as per local researcher's advice

Q12a: ask "Mc Donald's, Starbucks Coffee" in every country (They are the 'icons' of globalisation). For the other two items, rely on the local collaborator's advice. The third and fourth items may be Pizza Hut or KFC, which may nevertheless be absent in some countries. Try

whenever possible to ensure varieties in the type of food provided in the restaurants making up the examples. It is very difficult to find non-US food chains.

Q12b: Ask “Coca-Cola, Nestlé” in every country. Third example as per local researcher’s advice. Possible (non-US) items are Dannon (for US) or Danone, and Nescafè.

Q12c Ask Nike and Zara in each country. Third example as per local researcher’s advice. This may for instance be Adidas for the US (a German brand), or Levi’s for other countries.

NB: You may find other examples of transnational corporations at the Businessweek Top 100 global brands scoreboard (http://bwnt.businessweek.com/brand/2005/) or on the UNCTAD ranking of TNCs (http://www.unctad.org/Templates/Page.asp?intItemID=2443&lang=1)

Q32: as per Q84 (Pew), or local researcher’s advice

Q36: as per local researcher’s advice.

Q38: provide income categories considering the deciles of the income distribution in your country. Do not compute such categories at the current exchange rate between your currency and the USD, as that will probably make comparisons impossible.   Use conversions via purchasing power parity and the consensus of local experts.

SM.5: Further notes on construction of the IGI index

Questionnaire Question Number	Code	Belongs to participation index?	Notes
Q1 a-j	Pho/ Mob/ Internet/ Email/ Cred/ Post/ TV/ TVs/ Radio/ Fax	Yes	Q4 (Never) and Q5 (I do not own/have access, etc) are merged to form the lowest level in the 4-point participation to globalisation scale. Only Q5 counts towards the exposure index.<br>The participation index is formed as follows:<br>Answer 4,5 = 0<br>Answer 3 = 1/3<br>Answer 2= 2/3<br>Answer 1 = 1<br>NB: g and h have been merged, as typically having access to a satellite TV also implies access to a terrestrial TV.

N.	Code	Belongs to participation index?	Notes
Q2a-e	PhoLoc/<br>PhoNat/<br>PhoGlob/<br>PhoNot/<br><br>MobLoc/<br><br>MobNat/<br><br>MobGlob/<br><br>MobNot/<br><br>EmailL/<br><br>EmailN/<br><br>EmailG/<br><br>EmailNot/<br><br>PostLoc/<br><br>PostNat/<br><br>PostGlob/<br><br>PostNot/<br><br>FaxLoc/<br><br>FaxNat/<br><br>FaxGlob/<br><br>FaxNot	Yes	If answer in the previous question was“5”, and the answer to Q2 was different from 4 (does not apply) then do record the answer to Q2. If instead the answer has been “4” (=Never), then drop it as inconsistent.<br><br>Construct a participation index, either taking into account the broader level of connection, or constructing a weighted average.<br><br>Ex1:<br><br>- index = 1 if answer 3 (other countries) has been given<br><br>- index = 2/3 if answer 2 (other parts of my country) has been given and answer 3 has not been given<br><br>- index = 1/3 if answer 1 (local area) has been given and answers 2 and 3 have not been given<br><br>- index = 0 if answer is 4 (or answer 5or 4 in Q1).<br><br>Ex2: 1X(Indicator of whether answer 3 has been given) + 2/3X(Indicator of whether answer 2 has been given)+ 1/3X(Indicator of whether answer 1 has been given)
Q3a-c	TravNat/<br>TravCon/<br><br>TravGlob	Yes	

N.	Code	Belongs to participation index?	Notes
Q4a-d	GlobWarm/ Disease/<br><br>Court/<br><br>Gap	Yes	If Answer=5, then that counts towards the exposure index, but is a missing value for the participation index.<br><br>Participation index is formed as follows:<br><br>Answer 1 = 0<br><br>Answer 2 = 1/3<br><br>Answer 3= 2/3<br><br>Answer 4 = 1
Q5a-d	AidDis/<br><br>AidPov/<br><br>Sport/<br><br>IntEvent	Yes	In this case, the participation index is simply:<br><br>Answer 1= 1<br><br>Answer 2=0
Q6a-e	W_TV/<br><br>W_News/<br><br>R_News/<br><br>R_Mag/<br><br>Book/<br><br>Music	Yes	Q4 (Never) and Q5 (I do not own/have access, etc) are merged to form the lowest level in the 4-point participation to globalisation scale. Only Q5 counts towards the exposure index.<br><br>The participation index is formed as follows:<br><br>Answer 4,5 = 0<br><br>Answer 3 = 1/3<br><br>Answer 2= 2/3<br><br>Answer 1 = 1

7a-d	UN/<br>MNC/<br>IntOrg/<br>NGO/	Yes	If Answer=5, then that counts towards the exposure index, but is a missing value for the participation index.<br>Participation index is formed as follows:<br>Answer 1 = 0<br>Answer 2 = 1/3<br>Answer 3= 2/3<br>Answer 4 = 1
**N.**	**Code**	**Belongs to participation index?**	**Notes**
8a-d	ContrUN/<br>IntAgr	Yes	Ditto
Q9	MultComp	Yes	In this case, the participation index is simply:<br>Answer 1 = 1<br>Answer 2 =0
10	Car	Yes	Participation index is formed as follows:<br>Answer 4 (I do not own a car) = 0<br>Answer 1 (In my country) = 1/2<br>Answer 2 (In a different country) = 1<br>Answer 3 = Missing value<br>NB: To be confirmed

11ba-c	RestO/<br><br>FoodO/<br><br>ClotO	Yes	If answer hasn't been a yes to corresponding 11aX question, then drop answer, unless answer is 4. Moreover, consider answers 2 and 3 in A11aX as lowest level in the participation scale.<br><br>That is:<br><br>If answer =4, or 2 in Q11ab, then 0<br><br>If answer =3, then 1/3<br><br>If answer =2, then 2/3<br><br>If answer =1, then 1

N.	Code	Belongs to participation index?	Belongs to exposure index?	Notes
12ba-c	RestMNCO/ FoodMNCO/ ClotMNCO	Yes	No	If answer hasn't been a yes to corresponding 12aX question, then drop this answer, unless answer is 4. Moreover, consider answers 2 or 3 in 12aX as lowest level in the participation scale.<br>That is:<br>If answer =4, or 2 and 3 in Q12aa, then 0<br>If answer =3, then 1/3<br>If answer =2, then 2/3<br>If answer =1, then 1
13	ForProd	Yes	No	Participation index is formed as follows:<br>Answer 2 (I deliberately avoid…)= 0<br>Answer 3 (It makes no difference) = 1/2<br>Answer 1 (I deliberately seek them out) = 1
14a	ForCurr	Yes	No	In this case, the participation index is simply:<br>Answer 1 = 1<br>Answer 2 =0
14b	Bank	Yes	No	Ditto
14c	Invest	Yes	No	Ditto; Disregard Q14 in S. Africa since it is illegal to hold foreign investments.
14ba	Dollar	Yes	No	Ditto (this only applies to Argentina).
14bb	Euro	Yes	No	Ditto
14bc	Other_Curr	Yes	No	Ditto

N.	Code	Belongs to participation index?	Belongs to exposure index?	Notes
Q15	ForLang	Yes	No	Participation index is formed as follows:<br>Answer 1 = 0<br>Answer 2 = 1/3<br>Answer 3= 2/3<br>Answer 4 = 1
Q16b	FriendFo	Yes	No	Participation index is formed as follows:<br>Answer 1 = 0<br>Answer 2 = 1/2<br>Answer 3= 1

N.	Code	Belongs to participation index?	Notes
Q17b	FriendEt	Yes	Participation index is formed as follows:<br>Answer 1 = 0<br>Answer 2 = 1/2<br>Answer 3= 1
Q18	Trust	No	
Q19	Fair	No	
Q20	Helpful	No	
Q21a-24c	A_Local-…<br>L_Global	No	
Q25a-m	Socwel/ Church/ Educ/ Labor/ Polit/ DCS/ Conserv/ Profass/ Youth/ Sports/ Women/ Peace/<br>Health	No	
Q26a-m	SocwelL/<br>SocwelN/<br>SocwelG<br>…<br>HealthL/<br>HealthN/<br>HealthG	Yes	Keep this answer only if answer to the corresponding question in 25 has been 3 (I belong).<br>Construct an index of participation analogous to Q2. Answers 1 or 2 to the corresponding question in Q25 may be taken as the lowest level of the participation index.
Q27a-c	Culture/ WayLife/ Entry	No	
Q28a-b	Conn_EB/ Conn_CM	No	

Q29	SatLife	No	
Q30	Gender	No	
**N.**	**Code**	**Belongs to participation index?**	**Notes**
Q31	Year	No	
Q32	Educ	No	
Q33a-d	Country/ Count_P/ FamRes/ YouRes	Yes	In this case, the participation index is simply:<br>Answer 1= 1<br>Answer 2=0
Q34	Ethn	No	
Q35	Status	No	
Q36	Employm	No	
Q36other	Employ_O	No	
Q37NB	Occ_NB	No	
Q38	Income	No	
